# Deep Learning for Deep Chemistry: Optimizing the Prediction of Chemical Patterns

**DOI:** 10.3389/fchem.2019.00809

**Published:** 2019-11-26

**Authors:** Tânia F. G. G. Cova, Alberto A. C. C. Pais

**Affiliations:** Coimbra Chemistry Centre, CQC, Department of Chemistry, Faculty of Sciences and Technology, University of Coimbra, Coimbra, Portugal

**Keywords:** machine-learning, deep-learning, optimization, models, molecular simulation, chemistry

## Abstract

Computational Chemistry is currently a synergistic assembly between *ab initio* calculations, simulation, machine learning (ML) and optimization strategies for describing, solving and predicting chemical data and related phenomena. These include accelerated literature searches, analysis and prediction of physical and quantum chemical properties, transition states, chemical structures, chemical reactions, and also new catalysts and drug candidates. The generalization of scalability to larger chemical problems, rather than specialization, is now the main principle for transforming chemical tasks in multiple fronts, for which systematic and cost-effective solutions have benefited from ML approaches, including those based on deep learning (e.g. quantum chemistry, molecular screening, synthetic route design, catalysis, drug discovery). The latter class of ML algorithms is capable of combining raw input into layers of intermediate features, enabling bench-to-bytes designs with the potential to transform several chemical domains. In this review, the most exciting developments concerning the use of ML in a range of different chemical scenarios are described. A range of different chemical problems and respective rationalization, that have hitherto been inaccessible due to the lack of suitable analysis tools, is thus detailed, evidencing the breadth of potential applications of these emerging multidimensional approaches. Focus is given to the models, algorithms and methods proposed to facilitate research on compound design and synthesis, materials design, prediction of binding, molecular activity, and soft matter behavior. The information produced by pairing Chemistry and ML, through data-driven analyses, neural network predictions and monitoring of chemical systems, allows (i) prompting the ability to understand the complexity of chemical data, (ii) streamlining and designing experiments, (ii) discovering new molecular targets and materials, and also (iv) planning or rethinking forthcoming chemical challenges. In fact, optimization engulfs all these tasks directly.

## Introduction

Patterns are ubiquitous in Chemistry. From the crystalline structures of solid forms to the branched chains of lipids, or the complex combinations of functional groups, chemical patterns determine the underlying properties of molecules and materials, essential to address important issues of societal concern. Artificial Intelligence (AI), and machine learning (ML) in particular, are committed to recognizing and learn from these patterns (Mitchell, 2014; Rupp, 2015; Goh et al., 2017; Li et al., 2017; Butler et al., 2018; Fleming, 2018; Gao et al., 2018; Kishimoto et al., 2018; Popova et al., 2018; Aspuru-Guzik et al., 2019; Elton et al., 2019; Gromski et al., 2019; Mater and Coote, 2019; Schleder et al., 2019; Venkatasubramanian, 2019).

Recent evidence on the most interesting and challenging prospects for accelerating discoveries in various chemistry fields, reported under “Charting a course for chemistry” (Aspuru-Guzik et al., 2019), indicate that the terms often used by the scientific community for describing the future trends in their field of research include “big data,” “machine learning,” and “artificial intelligence.”

It is recognized that ML is already boosting computational chemistry, at different levels. Different aspects have been affected, and it is not easy to summarize developments in a consistent way. In what follows, the main areas in which ML has been employed are enumerated. These are extracted from recent contributions, that can be regarded as complementary and providing an overall perspective of the applications. These include different approaches for (i) understanding and controlling chemical systems and related behavior (Chakravarti, 2018; Fuchs et al., 2018; Janet et al., 2018; Elton et al., 2019; Mezei and Von Lilienfeld, 2019; Sanchez-Lengeling et al., 2019; Venkatasubramanian, 2019; Xu et al., 2019; Zhang et al., 2019), (ii) calculating, optimizing, or predicting structure-property relationships (Varnek and Baskin, 2012; Ramakrishnan et al., 2014; Goh et al., 2017; Simões et al., 2018; Chandrasekaran et al., 2019), density functional theory (DFT) functionals, and interatomic potentials (Snyder et al., 2012; Ramakrishnan et al., 2015; Faber et al., 2017; Hegde and Bowen, 2017; Smith et al., 2017; Pronobis et al., 2018; Mezei and Von Lilienfeld, 2019; Schleder et al., 2019), (iii) driving generative models for inverse design (i.e., produce stable molecules from a set of desired properties) (White and Wilson, 2010; Benjamin et al., 2017; Kadurin et al., 2017; Harel and Radinsky, 2018; Jørgensen et al., 2018b; Kang and Cho, 2018; Li et al., 2018b; Sanchez-Lengeling and Aspuru-Guzik, 2018; Schneider, 2018; Arús-Pous et al., 2019; Freeze et al., 2019; Jensen, 2019), (iv) screening, synthesizing, and characterizing new compounds and materials (Ahneman et al., 2018; Coley et al., 2018a; Granda et al., 2018; Segler et al., 2018; Li and Eastgate, 2019), (v) improving catalytic technologies and analytical tools (Li et al., 2017; Gao et al., 2018; Huang et al., 2018; Durand and Fey, 2019; Freeze et al., 2019; Schleder et al., 2019), (vi) developing quantum algorithms for molecular simulations, and (vii) progressing quantum sensing (Ramakrishnan et al., 2014; Ramakrishnan and Von Lilienfeld, 2017; Xia and Kais, 2018; Ahn et al., 2019; Christensen et al., 2019; Mezei and Von Lilienfeld, 2019; Zaspel et al., 2019; Zhang et al., 2019), just to name a few examples. In fact, Chemistry is a data-rich area, encompassing complex information which is often unstructured and poorly understood.

Deep learning (DL) approaches can also be particularly useful to solving a variety of chemical problems, including compound identification and classification, and description of soft matter behavior (Huang et al., 2018; Jha et al., 2018; Jørgensen et al., 2018b; Popova et al., 2018; Segler et al., 2018; Zhou et al., 2018; Chandrasekaran et al., 2019; Degiacomi, 2019; Elton et al., 2019; Ghosh et al., 2019; Mater and Coote, 2019; Matsuzaka and Uesawa, 2019; Xu et al., 2019).

The design of generalized cause/effect models, and the scaling-up of the contributions that are being made, containing high-dimensional data, and following the open-science basis (i.e., completely accessible, with precise metadata and practical formats) are critical challenges, that may, however, facilitate the routine implementation of data mining in chemistry and expedite new discoveries.

The amount and quality of chemical data generated by experiments and simulations have been the mainstay of the new data-driven paradigm, that establishes the bridge between theory, experiment, computation, and simulation.

This review describes, in a critical and comprehensive way, relevant contributions carried out recently and involving the development of chemistry ML approaches. An exhaustive account of the theoretical foundations and applications published in the early years of AI and ML in Chemistry falls beyond the scope of this review. The reader is referred to Lecun et al. (2015), Coveney Peter et al. (2016), Goh et al. (2017), Elton et al. (2019), Gromski et al. (2019), and Mater and Coote (2019) for a full description of these efforts.

Until 10 years ago, only a few 100 studies on the use of ML in Chemistry were published, resulting from the contributions made over four decades. In 2018, ca. 8,000 articles in the Web of Science database included these keywords, corresponding to an increase in ca. 35% for just one decade. In this review, there is room to mention only a small fraction of these applications.

Despite the increasing number of works on the topic, the models proposed and practices carried out by chemists are entailing serious concerns (Chuang and Keiser, 2018a). Several technical challenges, pitfalls, and potentials of ML, and also the reliability of the results, have been discussed by some authors (Ahneman et al., 2018; Chuang and Keiser, 2018a,b; Estrada et al., 2018) corroborating some critical remarks on the fragility of purely data-based approaches (Microsoft, 2018). “If data can speak for themselves, they can also lie for themselves.” This reflects the need for an in-depth understanding of chemical patterns, data-driven and theory-driven models, and algorithms, before their application.

Although significant progress has been made connecting specific neural network predictions to chemical input features, understanding how scientists should analyze and interpret these models to produce valid and conclusive assumptions about the system under study, still remains to be fully defined.

### Co-occurring Machine-Learning Contributions in Chemical Sciences

Scientific production covering ML-based approaches for dealing with chemical patterns has increased exponentially in recent years. However, the establishment and understanding of holistic, or macro insights on the major research trends in Chemistry sub-fields, are critical tasks. The challenge relies on how the analysis of these sub-fields, with thousands published works, reveals the most prominent applications supported by ML approaches (Butler et al., 2018; Chmiela et al., 2018; Chuang and Keiser, 2018a; Coley et al., 2018a; Gao et al., 2018; Lo et al., 2018; Panteleev et al., 2018; Xia and Kais, 2018; Ceriotti, 2019; Chan et al., 2019; Christensen et al., 2019; Gallidabino et al., 2019; Häse et al., 2019; Iype and Urolagin, 2019; Mezei and Von Lilienfeld, 2019; Schleder et al., 2019; Stein et al., 2019a; Wang et al., 2019).

**Figure 1:**
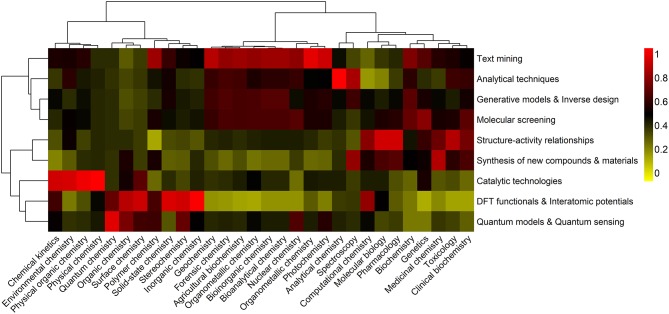
A holistic view of ML-based contributions in Chemistry. The clustering heatmap displays the relative counts of ML outcomes, within each area of Chemistry (organic, inorganic, analytical, physical, and biochemistry), in the 2008–2019 (30 June) period. Data are expressed as fractions of the highest number of publications, including articles, reviews and books, containing specific co-occurring keywords, and following a standard normalization procedure. Hierarchical clustering with Euclidean distances and Ward linkage was performed on both Chemistry sub-fields and type of application. Co-occurrences are colored using a yellow-to-red color scheme. Highest and lowest relative contributions correspond to 1 (red) and 0 (yellow) values, respectively.

In Figure 1 an overview of the information generated during the last decade and ranked in the research domain of “Science Technology” of the Web of Science database, is presented.

The purpose of assessing the different facets of ML in Chemistry across the respective sub-fields is 3-fold: (i) to be able to quickly identify areas that have benefited most from the development and implementation of ML approaches, and those that still lack of such an optimization, as evidenced by the type of outcome, (ii) to identify the most relevant ML outcomes in each sub-field, and (iii) to assess the dynamics of ML outcomes over the 2008–2019 period and how these are related, giving rise to relevant research trends.

An extensive literature search on ML contributions in 30 Chemistry sub-fields is carried out, using a global set of 270 co-occurring keywords, each composed of three main terms, *machine learning, type of outcome* and the *sub-field* in which they co-occur (e.g., first co-occurrence: *Machine learning* AND *Quantum chemistry* AND *Quantum models*, second co-occurrence: *Machine learning* AND *Medicinal Chemistry* AND *Molecular screening*). A total of 5,279 contributions (including books, articles, reviews, editorials and letters) on ML in Chemistry, with 81,248 citations, and published between 2008 and June 30, 2019, are found in the worldwide Web of Science database, corresponding to a 4-fold increase over the previous four decades. Considering the compiled data and the selected Chemistry fields (organic, inorganic, physical, analytical, and biochemical), nine different ML outcomes embracing the most frequent chemical challenges are defined, including (i) text mining and system description, (ii) quantitative structure-activity/property relationships, (iii) DFT functionals and interatomic potentials, (iv) generative models and inverse molecular design, (v) molecular screening, (vi) synthesis/characterization of new compounds and materials, (vii) catalytic technologies, (viii) analytical techniques, and (ix) quantum models, algorithms, and quantum sensing. Note how these have a strong relation with the seven overall applications presented above (i–vii).

The heatmap represented in Figure 1 reflects the impact of each type of ML outcomes on Chemistry sub-fields. The analysis of co-occurring keywords is thus performed in order to find the number of publications that appeared simultaneously in the selected sub-field. This relation is established with greater or lesser impact depending on the frequency of each set of keywords in the selected time-span.

The natural clusters generated from the most important co-occurring relationships are also identified. Considering the dendrogram for the Chemistry sub-fields, it can be observed that these are organized in two main groups, which discriminates, in general, classical Chemistry sub-fields (organic, inorganic, and physical) from analytical and biochemical sub-fields. This structure suggests a significant similarity in the type of ML outcomes within each group. Group 1 have benefitted from a significant production on catalytic technologies, DFT functionals and interatomic potentials, quantum models and quantum sensing. The most representative ML outcomes in group 2 are associated to text mining, analytical techniques, generative models and inverse design, molecular screening, structure activity relationships, and synthesis of new compounds and materials. Examination of the similarity between the type of ML outcomes reveals that there are three main groups, corresponding to (i) text mining, analytical techniques, generative modes and inverse design, and molecular screening (group 1), (ii) structure-activity relationships and synthesis of new compounds and materials (group 2), and (iii) catalytic technologies, DFT functionals and interatomic potentials, and quantum models and quantum sensing (group 3).

Historically, researchers have introduced numerical approximations to Schrödinger's equation, and the popular DFT calculations in *ab initio* approaches. However, the computational cost inherent to these classical approximations have limited the size, flexibility, and extensibility of the studies. Larger searches on relevant chemical patterns, have been successfully conducted since several research groups have developed ML models and algorithms to predict chemical properties using training data generated by DFT, which have also contributed to the increase of public collections of molecules coupled with vibrational, thermodynamic and DFT computed electronic properties (e.g., Behler and Parrinello, 2007; Rupp et al., 2012; Behler, 2016; Hegde and Bowen, 2017; Pronobis et al., 2018; Chandrasekaran et al., 2019; Iype and Urolagin, 2019; Marques et al., 2019; Schleder et al., 2019).

Based on the heatmap it can be determined that groups of Chemistry sub-fields have similar, but distinct ML-based contributions.

The increase in chemical data and scientific documents has boosted data mining and text mining processes to manage the huge amount of chemical information and to extract useful and non-trivial knowledge in different scenarios (Krallinger et al., 2017).

It is interesting to inspect if certain ML outcomes are produced in combination with each other.

**Figure 2:**
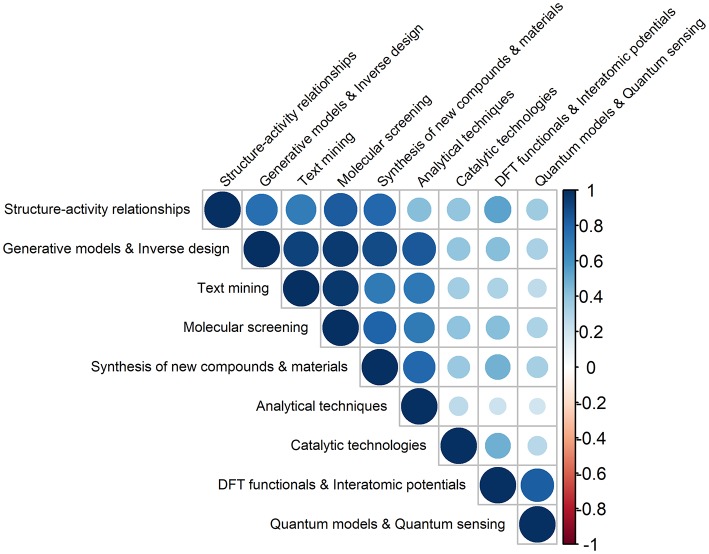
Pairwise Pearson correlations between the different types of ML outcomes in Chemistry, produced in the 2008–2019 (30 June) period (darker colors reflect stronger correlations).

In this context, the strongest correlation (0.97), shown in Figure 2, is observed between text mining and molecular screening, which is to be expected as a large number of molecules has been collected and screened systematically, by combining different text mining processes and chemoinformatics techniques (e.g., pharmacophore-based similarity and docking). These integrated approaches have allowed (i) extracting and collecting, in a systematic and high-throughput way, the available chemical and biological information from different sources (e.g., scientific documents) (Krallinger et al., 2017; Grzybowski et al., 2018), (ii) predicting activity based on chemical structure (Granda et al., 2018; Simões et al., 2018; Arús-Pous et al., 2019; Gromski et al., 2019; Lee et al., 2019; Li and Eastgate, 2019), and (iii) selecting promising molecular targets and candidates for further experimental validation (e.g., *in vitro* tests) (Ramakrishnan et al., 2014; Gupta et al., 2018; Segler et al., 2018; Brown et al., 2019; Elton et al., 2019; Li and Eastgate, 2019; Schleder et al., 2019; Xu et al., 2019).

Other strong correlations are found between generative models & inverse design and the two abovementioned ML applications, molecular screening (0.95) and text mining (0.93). This can be explained by the fact that many researchers have proposed machine learning frameworks based on a variety of generative models for modeling molecules, which differ in the respective model structure and in the selected input features (Kadurin et al., 2017; Gupta et al., 2018; Jørgensen et al., 2018b; Arús-Pous et al., 2019; Brown et al., 2019; Jensen, 2019; Xu et al., 2019).

Also relevant are the correlations between generative models and inverse design and synthesis of new compounds and materials (0.90), and between generative models and inverse design and analytical techniques (0.85). The former relation evidences the significant effort that has been made on applying ML models, in particular those based on accurate DL architectures, to find and select lead molecules (e.g., drugs), displaying desired properties (Varnek and Baskin, 2012; Mitchell, 2014; Rupp, 2015; Lo et al., 2018). These properties are to be translated into a more simplified information on the molecular structures, and encoded into the respective chemical fingerprint (i.e., a set of binary characteristics of molecules). The process continues with the screening of the available databases for finding molecules that possess similar fingerprints to the generated ones. Generative models and deep neural networks (DLNs) have thus allowed generating molecules and promising candidates for useful drugs, basically from scratch, making it possible to “design perfect needles instead of searching for a needle in a haystack” (White and Wilson, 2010; Benjamin et al., 2017; Gómez-Bombarelli et al., 2018; Harel and Radinsky, 2018; Kang and Cho, 2018; Li et al., 2018b; Merk et al., 2018; Nouira et al., 2018; Popova et al., 2018; Sanchez-Lengeling and Aspuru-Guzik, 2018; Schneider, 2018).

It is also observed that there are other ML contributions that are interrelated: structure activity relationships with (i) molecular screening and (0.84), (ii) synthesis/characterization of new compounds and materials (0.78), and (iii) generative models and inverse design (0.75), DFT functionals and interatomic potentials with quantum models and quantum sensing (0.83), and synthesis/characterization of new compounds and materials with analytical techniques (0.79).

Both generative models and analytical techniques have been extensively used in the qualitative/quantitative search of patterns underlying chemical systems (Elton et al., 2019; Ghosh et al., 2019; Stein et al., 2019a,b). It should be noted the use data from large repositories (e.g., Protein Data Bank and Cambridge Structural Database) and ML methods are not new (Hiller et al., 1973; Gasteiger and Zupan, 1993; Behler, 2016). The latter have been employed as classification tools in pioneering works, encompassing, for e.g., the analysis of spectra (Thomsen and Meyer, 1989), quantification of structure-activity relationships (QSARs) (Agrafiotis et al., 2002), and prediction of binding sites of biomolecules (Keil et al., 2004).

The range of ML applications is now quite extended as a result of a deep integration of ML in analytical, theoretical and computational chemistry. Despite of some initial skepticism in understanding the foundations and structure of ML methods, their use has been accelerated and maturated in recent years essentially due to their suitability to new applications and industry needs, including chemical and pharmaceutical sectors.

## Machine Learning For Optimization: Challenges and Opportunities

Designing models from chemical observations to study, control, and improve chemical processes and properties is the basis of optimization approaches. The understanding of chemical systems, and the respective underlying behavior, mechanisms and dynamics, is currently facilitated by the development of descriptive, interpretative, and predictive models, i.e., approximations that represent the target system or process. Applications of such models have included the (i) optimization of reaction parameters and process conditions, e.g., changing the type of reagents, catalysts, and solvents, and also varying systematically, concentration, addition rate, time, temperature, or solvent polarity, (ii) suggestion of new reactions based on critical functional groups, (iii) prediction of reaction/catalyst design, and optimization of heterogeneous/homogeneous catalytic reactions, (iv) acceleration and discovery of new process strategies for batch reactions, (v) establishment of trade-offs in the reaction rate and yield of organic compounds, (vi) description and maximization of the production rate and conversion efficiency of chemical reactions, (vii) prediction of the potential toxicity of different compounds, and also the (viii) rational design of target molecules and guided exploration of chemical space (Kowalik et al., 2012; Houben and Lapkin, 2015; Houben et al., 2015; Zielinski et al., 2017; Häse et al., 2018; Min et al., 2018; Zhou et al., 2018; Ahn et al., 2019; Choi et al., 2019; Gromski et al., 2019; Matsuzaka and Uesawa, 2019).

ML provides the tools to scrutinize and extract useful information to be employed in modeling and system-solving solutions (Artrith and Urban, 2016; Ward and Wolverton, 2017). In Chemistry domains, researchers have had access to multidimensional data of unprecedented scale and accuracy, that characterize the systems/processes to be modeled. A collection of different examples of optimization based on ML approaches can be found in Kowalik et al. (2012); Houben and Lapkin (2015); Houben et al. (2015); Cortés-Borda et al. (2016); Wei et al. (2016); Benjamin et al. (2017); Ahneman et al. (2018); Gao et al. (2018); Granda et al. (2018); Min et al. (2018); Ahn et al. (2019); Elton et al. (2019); Matsuzaka and Uesawa (2019).

Specifically, ML contributions have involved a variety of systems including drugs (Griffen et al., 2018), polymers (Li et al., 2018a), polypeptides (Grisoni et al., 2018; Müller et al., 2018), energetic materials (Elton et al., 2018), metal organic frameworks (He et al., 2018; Jørgensen et al., 2018a; Shen et al., 2018), and organic solar cells (Jørgensen et al., 2018a).

Advances in analytical methods, laboratory equipment and automation have rapidly improved the performance of experimental procedures (e.g., miniaturizing experiments for reactions, and connecting analytical instruments to advanced software based on decision-making algorithms and optimization tools) (Stevens et al., 2010; Smith et al., 2011; Richmond et al., 2012; Houben and Lapkin, 2015). The implementation of ML-based approaches have allowed developing innovative capabilities, such as cost-effective experiments, advanced algorithms for automation, and designing of experiments, chemoinformatics tools for dealing with high-dimensional analytical data, and accelerated *in situ*/in line analysis of chemical transformations (e.g., polymerization reactions, heterogeneous catalytic processes, aggregation of nanoparticles) (Houben and Lapkin, 2015; Häse et al., 2018).

However, there are critical challenges that ML in Chemistry must face, including the control of experiments, the detailed description of chemical space, the flexibility and generalization of models, robustness, and accuracy of predictions, and the establishment of effective cross-disciplinary collaborations (Montavon et al., 2013; Hansen et al., 2015; Kishimoto et al., 2018; Smith et al., 2018a).

A clear definition of ML, as well as the distinction from other purely mathematical regression methods is not straightforward, and can be associated to some degree of arbitrariness (Behler, 2016). Standard ML methods include, artificial neural networks, support vector machines, and Gaussian processes, which have contributed to the rational design of compounds and materials, and to the improvement of computational frameworks (Goh et al., 2017; Mater and Coote, 2019). The latter have been applied for e.g., in QSAR models and drug design (Kadurin et al., 2017; Chen et al., 2018; Fleming, 2018; Green et al., 2018; Gupta et al., 2018; Li et al., 2018b; Lo et al., 2018; Popova et al., 2018; Simões et al., 2018) aiming at identifying systems, molecules and materials with optimal properties (e.g., conductivity, aqueous solubility, bioavailability, bioactivity, or toxicity) (Kadurin et al., 2017; Freeze et al., 2019). This can be made via extensive searches, in large databases, of latent relationships between the atomic structures. The structures, can thus be encoded using multiple descriptors, and target properties.

The possibilities of applying ML for optimization in Chemistry are endless. There are studies focused on ML approaches for inferring on the optimized geometry of a system (Zielinski et al., 2017; Venkatasubramanian, 2019), and finding minima on complex potential energy surfaces (Chen et al., 2015; Chmiela et al., 2018; Kanamori et al., 2018; Xia and Kais, 2018; Hughes et al., 2019), such as those of large water clusters (Bose et al., 2018; Chan et al., 2019).

The most innovative aspects of ML in Chemistry are related to the availability of large volumes of theoretical data (e.g., electrostatic energy contributions in force fields, atomic charges, structural properties, and representations of the potential energies), obtained from automatic and accurate electronic structure calculations (Behler, 2016).

However, the intricate nature of the configuration space and its exponential dependence on system size and composition, have hampered the screening of the entire set of candidate structures directly by electronic structure calculations (Behler, 2016; Welborn et al., 2018).

### Signs of Controversy

Despite the usefulness of ML approaches being indisputable, with the promise to modernize molecular simulations, synthesis, materials science, and drug discovery, the respective endorsement and practical aspects in some chemical sub-fields is far from consensual (Ahneman et al., 2018; Chuang and Keiser, 2018a,b).

Ten years ago, there were only a few publications on applications of ML in Chemistry, but currently there are thousands of published works. The controversy has highlighted the potential (instructive) pitfalls of some practices using ML. It has been argued that ML algorithms may lead to overestimated performances and deficient model generalizations, due to their sensitivity to the presence of maze-like variables and experimental artifacts (Chuang and Keiser, 2018a). For instance, Ahneman et al. (2018) have recently designed a ML model to predict yields of cross coupling reactions with high accuracy, containing isoxazoles, as reaction inhibitors, which were incorporated for assessing the robustness of the reaction. Input data for the proposed algorithm included yields and reagent parameters of 3,000 reactions, such as NMR shifts, dipole moments, and orbital energies. The most significant features of the proposed algorithm were found to be the descriptors of additives. However, the experimental design of this original work has been contested by Chuang and Keiser (2018b), who warned for potential artifacts associated to the original work. These authors demonstrated that the model also identified reaction additives as the descriptors displaying the greatest impact on the reactions, suggesting that high additive feature contributions cannot be discriminated from the hidden structure within the dataset, i.e., the procedure of the original paper was not sufficient for establishing isoxazole additives as the most important descriptors (Chuang and Keiser, 2018b). A meticulous preprocessing of input data and validation of the model hypothesis was then suggested. The Y-randomization test in the original work was taken into account just the information rooted in the structure of the data set, irrespective to the intended outcome. The classical approach based on multiple hypotheses to assess alternative descriptions of the performance of the ML model was implemented (Chuang and Keiser, 2018b). The effect of different reaction parameters (e.g., additives, catalyst, and aryl halide) in an extensive combinatorial layout generated over several independent reactions was duly explored, providing the underlying structure of the data (Chuang and Keiser, 2018b).

An alternative assumption considering that ML algorithms deal with patterns within the experimental design, instead of learning from the most relevant chemical features was therefore investigated. It was concluded that ML is prone to explore data irrespective to their size and structure. This aspect was illustrated by extracting and replacing the chemical features (e.g., electrostatics, NMR shifts, dipole moments) from each molecule with random (Gaussian distributed) numeric strings. It was shown that the predictions were similar to the original ones. Chuang and Keiser (2018a) have also introduced technical and conceptual standpoints, including the use of adversarial controls to evaluate the predictive performance of ML models, focusing on the design of rigorous and deliberated experiments, ensuring accurate predictions from suitable and significant models (Chuang and Keiser, 2018a). By revising the original information, a number of variations of the test sets was introduced by Estrada et al. (2018) for assessing the performance of predictions, considering alternatives to the random-forest model. It was therefore demonstrated that ML models are in fact quite sensitive to such imposed features, and the reagent-label models are relevant representations of the data set and useful for comparing performances in generalization assessments.

The original assumptions regarding the significance and validity of the random-forest (chemical-feature) model to describe important and general chemical features were also confirmed (Estrada et al., 2018).

A lesson that chemists may draw from such constructive discussions is that as the size of the data set increases, the performance of ML models also increases, but with the possibility of obtaining unexpected results and irrelevant patterns, as the rules for ML algorithms to detect and deal with potential technical and conceptual gaps are not well-established. Specifically, the description of chemical reactivity underlying a data set is required in order to ensure the reaction prediction, by using data and reagent-label models to evaluate the scope and restraints of chemical characterization.

ML provides new opportunities to increase the quality and quantity of chemical data, which are essential to promote optimization, implementation of rational design and synthetic approaches, prioritization of candidate molecules, decision-making, and also for guiding of innovative ideas.

### Deep Learning, Deep Chemistry

In this section, an introductory overview into the core concepts of DL, and DLNs is provided. Focus is given to the unique properties of DL, that distinguish these algorithms from traditional machine learning approaches, with emphasis on chemical applications rather than providing theoretical and mathematical details.

ML is a branch of computer science dedicated to the development of algorithms capable of learning and making decisions on complex data (Samuel, 1959; Mitchell, 1997). This learning process involves specific tasks that are commonly classified in (i) supervised learning, for establishing the relationship between input and output data (e.g., linear regressions and classification techniques), (ii) unsupervised learning, for finding hidden patterns or features in data, without any previous information on such characteristics and interrelations (e.g., clustering and dimension reduction techniques), and (iii) reinforcement learning, for performing a particular task through repeated dynamic interactions e.g., optimization of molecules (Zhou et al., 2018) and chemical reactions (Zhou et al., 2017).

Deep learning is a fast-moving sub-area of ML, focused on sophisticated learning and extrapolation tasks, fostered by the wide range of chemistry literature, open-source code, and datasets (Goh et al., 2017).

The ability of DL to establish the relevant phenomena, expedite chemical reactions, and predict relevant properties, optimal synthesis routes, solve critical analytical uncertainties, and reduce costs and resources, is invaluable in Chemistry. Its success in modeling compound properties and reactions, depends, among other aspects, on the access to comprehensive, historical repositories of published chemical data (Venkatasubramanian, 2019).

There are barriers to be surpassed, including cleaning data, production of meaningful and accurate chemical information (free of bias), lack of standardization of chemical data, expertise and familiarity with ML and DL in chemistry sectors, and also lack of collaboration opportunities) (Mater and Coote, 2019).

The majority of DL algorithms currently developed are based on artificial neural networks (Lecun et al., 2015).

**Figure 3:**
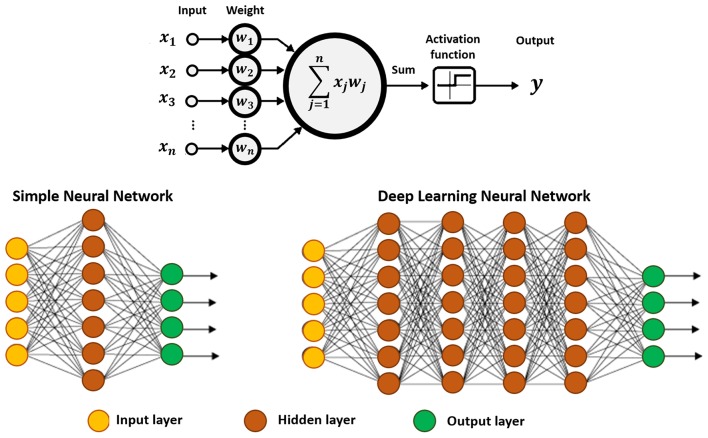
Schematic representation of an artificial neuron (top), and a simple neural network displaying three basic elements: input, hidden and output layers (bottom-left), and a deep neural network showing at least two hidden layers, or nodes (bottom-right). The calculation is performed through the connections, which contain the input data, the pre-assigned weights, and the paths defined by the activation function. If the result is far from expected, the weights of the connections are recalibrated, and the analysis continues, until the outcome is as accurate as possible.

DLNs are now a proving-ground for research in chemical sciences (Goh et al., 2017; Jha et al., 2018; Popova et al., 2018; Segler et al., 2018; Elton et al., 2019; Mater and Coote, 2019; Xu et al., 2019). Similarly to artificial neural networks, DLNs are produced to resemble the brain, in which the information passes through a series of interconnected nodes comparable to neurons (Lecun et al., 2015). Each node analyzes segments of information and transfer that information to adjacent nodes (see Figure 3).

The computational model consists of multiple hidden layers (in higher number comparing to more conventional approaches) which confer the ability of DLNs to learn from highly complex data and perform correlation and reduction. This means that the algorithm discovers correlated data, while discarding irrelevant information. Each layer combines information collected from the previous layer, and subsequently infers on the respective significance and send the relevant information to the next layer. The hidden term is used to represent layers that are not direct neighbors of the input or output layers.

The process allows constructing increasingly complex and abstract features, by adding layers and/or increasing the number of neurons per layer. However, the use of more than a single hidden layer requires determining error attributions and corrections to the respective weights. This is carried out via a backpropagation, i.e., a backward process starting from the predicted output, and back through the neural network (Goh et al., 2017). In this process a gradient descent algorithm is employed to determine the minimum in the error surface created by each respective neuron, when generating the output. Note that, this gradient descent approach is conceptually similar to the steepest descent algorithm implemented in classical MD simulations (Goh et al., 2017). The major difference lies on the use of an iterative process, in which an error function of the target output of the neural network is minimized, and the weights of the neurons are updated, instead of iteratively minimizing an energy function and updating atomic coordinates for each step.

A complete description of the main core concepts and architecture of DL applied to chemistry is given in Goh et al. (2017) and Mater and Coote (2019).

Other interesting reviews covering theoretical aspects (Goh et al., 2017), available descriptors and datasets, and also comparing model performances (Wu et al., 2017) have been published. Moreover, a wide range of ML applications, including drug design (Ekins, 2016; Chen et al., 2018; Fleming, 2018), synthesis planning (Coley et al., 2018a), medicinal chemistry (Panteleev et al., 2018), cheminformatics (Lo et al., 2018), quantum mechanical calculations (Rupp, 2015), and materials science (Butler et al., 2018) have been collected.

**Figure 4:**
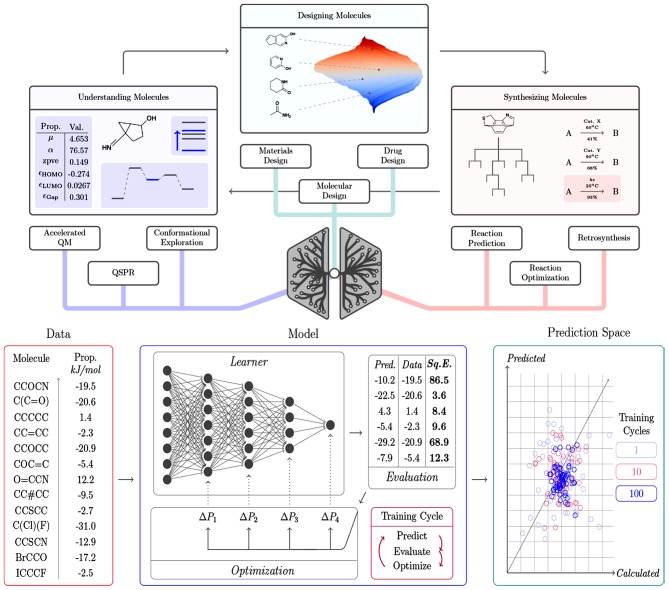
Overview of (top) the contribution of DL algorithms for solving different chemical challenges and the respective tasks, and (bottom) the general components of a DL framework, including the input data, the learning model able to interpret the data and the prediction space, from which the model performance can be inspected. The model represents an optimization cycle containing interconnected components: prediction, evaluation, and optimization. Reprinted with permission from Mater and Coote (2019). Copyright (2019) American Chemical Society.

A summary of the main contributions of DL for solving relevant chemical challenges, as well as an illustration of the general components of a DL framework are presented in Figure 4.

DL algorithms are particularly attractive for accelerating discoveries in pharmaceutical, medicinal and environmental chemistry (El-Atta and Hassanien, 2017; Goh et al., 2017; Klucznik et al., 2018; Miller et al., 2018; Panteleev et al., 2018; Smith et al., 2018b; Wu and Wang, 2018; Molga et al., 2019), since they have made possible, for e.g., to simulate millions of toxic compounds and identify those compounds displaying target properties, safely, economically, and sustainably. These types of applications have been thoroughly revised in various publications and will not be further addressed in what follows [see for e.g., (Kadurin et al., 2017; Chen et al., 2018; Fleming, 2018; Green et al., 2018; Gupta et al., 2018; Li et al., 2018b; Lo et al., 2018; Panteleev et al., 2018; Popova et al., 2018; Smith et al., 2018b)].

DL is not only a cost-cutting effort, but also an innovative source of new perspectives.

## Cutting-Edge Applications

In recent years, ML has been evoked in chemistry-related tasks. The use of ML and, in particular, DL-based approaches across prediction of binding, activity and other relevant molecular properties, compound/material design and synthesis, as well as applications of genetic algorithms are highlighted in what follows.

Researchers in chemical sciences have started exploring the capabilities of ML using data collected from computations and experimental measurements. Data mining is traditionally adopted to explore high-dimensional data sets, in order to identify and establish relevant connections for the chemical features of compounds and materials.

Other more ambitious approaches, including quantum mechanics, which integrates physics-based computations (e.g., DFT) and ML methods in the search for novel molecular components, have also been implemented (Curtarolo et al., 2013).

Amongst the major achievements of DL in Chemistry, are the outstanding performances in predicting activity and toxicity, in the context of the Merck activity prediction challenge in 2012, and the Tox21 toxicity prediction challenge launched by NIH in 2014, respectively. In the former, DL was very successful in the competition outperforming Merck's internal baseline model. In the second challenge, DL models also achieved top positions (Goh et al., 2017).

Similarly to what happens to the majority of the modern computational chemists who no longer build their own code to perform MD simulations or quantum chemical calculations, due to the existence and availability of well-established software packages, DL researchers have also use several software packages for training neural networks including Torch, Caffe, Theano, and Tensorflow (Goh et al., 2017).

Apart from the influence of software improvements, the continuous growth of chemical data in public databases, such as PubChem and Protein Data Bank has also facilitated the raise of ML and DL applications in Chemistry, including quantum chemistry, property prediction and materials design, drug discovery, QSAR, virtual screening, and protein structure prediction (Goh et al., 2017; Christensen et al., 2019).

### Improving Computational and Quantum Chemistry

Computational chemistry is naturally a sub-field that has been increasingly boosted by the advances and unique capabilities of ML (Rupp et al., 2012; Ramakrishnan et al., 2014, 2015; Dral et al., 2015; Sánchez-Lengeling and Aspuru-Guzik, 2017; Christensen et al., 2019; Iype and Urolagin, 2019; Mezei and Von Lilienfeld, 2019; Zaspel et al., 2019).

Also, recent progresses have enabled the acceleration of MD simulations (atomistic and coarse-grained), contributing to increase knowledge on the interactions within quantum many-body systems and efficiency of DFT-based quantum mechanical modeling methods (Bartók et al., 2010, 2013; Behler, 2011a,b, 2016; Rupp et al., 2012, 2015; Snyder et al., 2012; Hansen et al., 2013, 2015; Montavon et al., 2013; Schütt et al., 2014; Alipanahi et al., 2015; Botu and Ramprasad, 2015b; De et al., 2016; Faber et al., 2016; Sadowski et al., 2016; Wei et al., 2016; Brockherde et al., 2017; Chmiela et al., 2017, 2018; Smith et al., 2017; Wu et al., 2017; Gómez-Bombarelli et al., 2018). This field is still in its infancy and have offered invaluable opportunities for dealing with a wide range of challenges and unsolved questions, including but not limited to model accuracy, interpretability, and causality.

For instance, the prediction of the refractive index of ionic liquids based on quantum chemistry calculations and an extreme learning machine (ELM) algorithm has been conducted (Kang et al., 2018). Specifically, 1,194 experimental data points for 115 ionic liquids at different temperatures were collected from more than 100 literature reports. Quantum chemistry calculations were performed for obtaining the structures and descriptors of the ionic liquids. The model was designed using a stepwise regression algorithm and the *R*^2^ and AARD% values were 0.841 and 0.855%, respectively. It was found that prediction of the refractive index was significantly affected by ionic liquid anions, comparing to the cations. Better performances were achieve using the ELM algorithm, with the *R*^2^ and AARD% values of 0.957 and 0.295%, respectively (Kang et al., 2018).

ML has also contributed for modeling the water behavior, shedding light on important phenomena related to water molecules interactions and the resulting density. Morawietz et al. (2016) have calculated ice's melting point from fundamental quantum mechanics, demonstrating the predictive power of ab initio MD simulations and highlighting the critical role of van der Waals forces (Morawietz et al., 2016). It was evidenced that ice occupies a larger volume than liquid water as hydrogen bonds display water molecules in a rigid 3D network. These hydrogen bonds weaken when ice melts, and water molecules approximate, becoming dense with an extreme value at 4°C (Morawietz et al., 2016). Note that these processes can also be rationalized resorting to *ab initio* MD approaches based on DFT; however, such calculations are associated to highly demanding computations. In addition to this, DFT approaches are not able to accurately reproduce minute but relevant van der Waals forces. The same authors have trained a neural network to reproduce DFT results with less computer power, and employed a previously-existing van der Waals correction. Water density changes, hydrogen bond network flexibility, and competition effects in terms of the nearest shell's contraction, after cooling, were explained based on the simulations (Morawietz et al., 2016).

One of the current challenges is to answer the question of whether chemical-physical properties, that often require quantum mechanics (e.g., dipole moments, binding and potential energies, and thermodynamics), can be represented and predicted by ML methods (Hansen et al., 2013, 2015; Montavon et al., 2013; Faber et al., 2016; Iype and Urolagin, 2019; Jaquis et al., 2019). Several attempts have been made on the topic with some successful examples (Rupp et al., 2012; Faber et al., 2017).

Rupp et al. (2012) have developed a model based on nuclear charges and atomic positions for predicting molecular atomization energies of various organic compounds. A matrix composed of molecular elements and configuration was built, describing the potential energy of each individual atom and the Coulomb repulsion between nuclear charges. A non-linear regression scheme was employed for solving and mapping the molecular Schrödinger equation.

The regression models were trained and compared to atomization energies calculated with hybrid DFT, transforming a 1-h run (on average) of hybrid DFT per each atomization energy into milliseconds using ML. Cross-validation over more than seven thousand organic molecules yielded a mean absolute error below 10 kcal/mol. The authors have trained the ML algorithm on a set of compounds in a database, comparing the respective matrices to determine differences between molecules, so as to develop a landscape of such differences. Based on the atomic composition and configuration, the unknown molecule can be positioned in the landscape and the respective atomization energy can be estimated by the contributions (weights) obtained from the differences between the unknown and all known molecules (Rupp et al., 2012).

More recently, the impact of selecting regressors and molecular representations on the construction of fast ML models of several electronic ground-state properties of organic molecules has also been investigated (Faber et al., 2017). The performance of each combination between regressor, representation, and property was evaluated with learning curves, which allowed reporting out-of-sample errors, as a function of the size if the training set (ca. 118 k molecules). The QM9 database (Ramakrishnan et al., 2014) was used for extracting the molecular structures and properties at the hybrid DFT level of theory, and included data on dipole moment, polarizability, enthalpies and free-energies of atomization, HOMO/LUMO energies and gap, heat capacity, zero point vibrational energy, and the highest fundamental vibrational frequency.

Several regression methods including linear models (Bayesian ridge regression and elastic net regularization), random-forest, kernel ridge regression, and neural networks (graph convolutions and gated graph networks) were tested. It was concluded that out-of-sample errors were strongly affected by the molecular properties, and by the type of representation and regression method. Molecular graphs and graph convolutions displayed better performances for electronic properties, while kernel ridge regression and histograms of dihedrals were suitable for describing energy-related properties [see Faber et al. (2017) for details on other relevant combinations]. Predictions based on the ML model for all properties have shown lower deviations from DFT (B3LYP) than the latter deviated from experiment. ML models displayed thus an improved prediction accuracy than hybrid DFT, since experimental or explicitly electron correlated quantum data were available.

In terms of drug development Brockherde et al. (2017) have developed a ML algorithm for predicting the behavior of molecules with potential to be used as pharmaceuticals and in the design of new molecules, able to enhance the performance of emerging energetic materials, including solar cells, battery technologies, and digital displays. The main goal was to identify the underlying patterns in the molecular behavior, by employing the ML algorithm for understanding atomic interactions within a molecule and using such information to predict new molecular phenomena.

**Figure 5:**
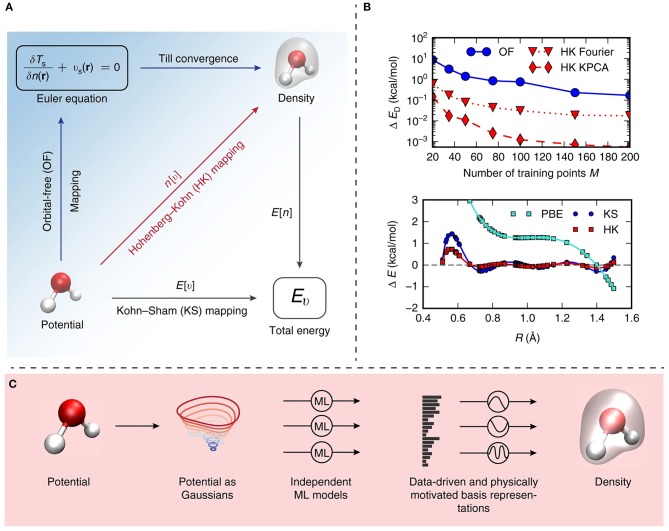
**(A)** Illustrative summary of the mappings proposed by Brockherde et al. (2017). *E*[*v*] is a conventional electronic structure calculation, i.e., Kohn–Sham density functional theory (KS-DFT) and is represented by the bottom vector. The ground-state energy is determined by solving KS equations given the external potential, *v*. *E*[*n*] corresponds to the total energy density functional. The Hohenberg–Kohn map n[v] (red vector) from external potential to its ground state density is also presented. **(B)** Top: graphical representation of the dependency of the energy error on the number of training points (M), for ML-OF and ML-HK, considering different basis sets for the one-dimensional problem. Bottom: errors in the Perdew-Burke-Ernzerhof (PBE) energies and the ML maps as a function of interatomic spacing, R, for H_2_ with M = 7. **(C)** Schematic illustration of the strategy for obtaining predictions based on the proposed machine learning Hohenberg–Kohn (ML-HK) map. Molecular geometry is represented by Gaussians, several independent Kernel ridge regression models allows predicting each basis coefficient of the density. The performance of data-driven (ML) and common physical basis representations for the electron density is assessed.

Specifically, the algorithm was created and trained on the basis of a small sample set of the molecule under study, and applied to simulate the intricate chemical behavior within selected molecules, including malonaldehyde. A directed learning of the density-potential and energy-density maps was conducted, as illustrated in Figure 5, and the first MD simulation of with a ML density functional on malonaldehyde was performed, allowing to describe the intramolecular proton transfer process (Brockherde et al., 2017).

**Figure 6:**
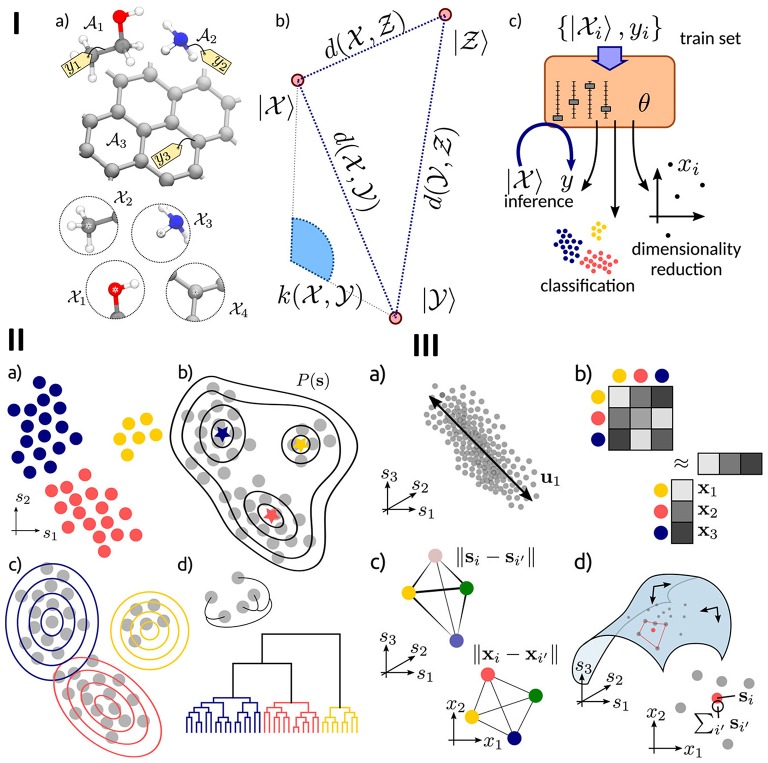
**(I)** Schematic representation of the main components of atomistic ML. **(a)** the inputs of the model are structures *A* or local environments *X*, **(b)** the mathematical representation of the inputs, based on vectors of features |*X* 〉, a measure of similarity d, or a kernel k, **(c)** the ML model, controlled by a series of parameters θ, and trained based on a set of inputs. **(II)** An overview of the clustering methods, including **(a)** a set of data points clustered according to their hidden common features, **(b)** a density-based clustering for identifying maxima in the probability distribution of inputs, **(c)** distribution-based clustering for finding a model of the data distribution based on the combination of clustering probabilities, and **(d)** hierarchical clustering for identifying natural clusters of the inputs. **(III)** Summary of dimensional reduction techniques, including principal component analysis (PCA) for establishing the most relevant subspace retaining the largest fraction of the input data variance, **(b)** a kernel-based method, **(c)** multidimensional scaling for reproducing in low dimension the similarity between high-dimensional data points. Reprinted with permission from Ceriotti (2019).

In more detail, one of the key tasks in atomistic modeling is the prompt and automated analysis of the simulation results, in order to provide a comprehensive understanding of the behavior of individual atoms and target collective properties. The main supervised and unsupervised machine-learning methods directed at classifying and coarse-graining of molecular simulations were recently summarized and discussed in Ceriotti (2019). A schematic overview of these methods, and also of a workflow reflecting the application of a ML scheme to an atomic-scale system is presented in Figure 6.

Also relevant is the development of improved molecular force fields, commonly used in MD simulations, using ML. On the other hand, the intrinsic operational aspects of MD simulations, in which the dynamic evolution of the chemical system is detailed in a fixed period of time, and for which interparticle forces and potential energies are often estimated using interatomic potentials, or molecular mechanics force fields, are perfectly suited for ML. In fact, some of the timesteps can be used as a training phase for estimating consecutive ones, assuming that each of the timesteps of MD simulation is strongly correlated with the preceding timestep and is adequate for sampling the phase space rapidly and accurately, allowing to estimate any meaningful property (Behler, 2016). MD simulations often sample abnormal, but probably relevant configurations, requiring the implementation of a decision tool for dealing with the unusual configuration, and from which ML may turn off and start learning (Botu and Ramprasad, 2015a; Smith et al., 2018a). These conditions have also been previously discussed and applied to *ab initio* MD (Botu and Ramprasad, 2015a).

In MD, the energies and forces for a vast number of atomic configurations are required, which can be obtained by performing the electronic structure calculations along the trajectory, or by evaluating the direct functional relation between the atomic configuration and the energy (Mansbach and Ferguson, 2015). This analytic expression, defined before running the simulation, is often recognized as a force field, an interatomic potential, or a potential-energy surface. Calculations of electronic structures are very demanding, even for DFT. DFT-based *ab initio* MD simulations are restricted to a few 100 atoms and shorter simulation times (Ahn et al., 2019).

The requirements for calculating ML potentials are very similar to conventional empirical potentials, and are duly discussed in Behler (2016). More recent conventional force fields are developed and validated for very specific systems, being limited by the functional form upon which they were constructed. On the other hand, despite requiring a training set, ML-based force fields are adaptive and more robust upon configurations not previously sampled (Botu and Ramprasad, 2015a). Furthermore, these force fields can be extended rapidly to different types of atoms and molecules, as they can learn and apply the physical laws, rather than starting from strarch (Botu et al., 2017).

Several improved force fields, and accurate predictions of thermodynamics and kinetics signatures, as well as their influence in molecular structures have been provided by performing ML-based atomistic and *ab initio* MD simulations. For instance, Chmiela et al. (2018) have incorporated spatial and temporal physical symmetries into a gradient-domain machine learning (sGDML) model for constructing flexible molecular force fields from high-level *ab initio* calculations, with a great potential to be used to improve spectroscopic accuracy in molecular simulations. The sGDML model was able to reproduce global force fields at quantum-chemical CCSD(T) level of accuracy and produced converged MD simulations with fully quantized electrons and nuclei (Chmiela et al., 2018).

**Figure 7:**
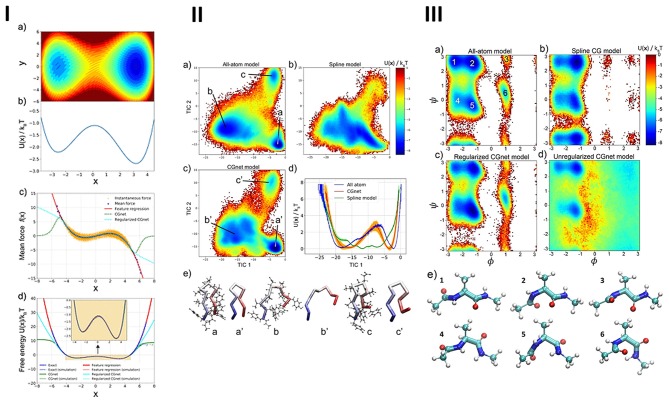
**(I)** Machine-learned coarse-graining of dynamics in **(a)** a two-dimensional potential, showing the **(b)** exact free-energy along x, comparison of **(c)** the instantaneous forces and the learned mean forces using feature regression and coarse-grained neural network models with the exact forces, and **(d)** the potential-of-mean-force along x, predicted by feature regression, and coarse-grained neural network models with the exact free energy. **(II)** Free-energy profiles and representative structures of alanine dipeptide simulated using all-atom and machine-learned coarse-grained models: **(a)** free-energy reference as a function of the dihedral angles, obtained from the histograms of all-atom simulations, **(b)** standard coarse-grained model using a sum of splines of individual internal coordinates, **(c)** regularized coarse-grained neural network models, **(d)** unregularized networks, **(e)** representative structures extracted from the free-energy minima, from atomistic simulation (ball-and-stick representation) and regularized coarse-grained neural network simulation (licorice representation). **(III)** Free-energy landscape of Chignolin for the different models, obtained from the **(a)** all-atom simulation, as a function of the first two TICA coordinates, **(b)** spline model, as a function of the same two coordinates used in the all-atom model, **(c)** coarse-grained neural network model, as a function of the same two coordinates. **(d)** Comparison of the one-dimensional free-energy profile as a function of the first TICA coordinate, reflecting the folding/unfolding transition, for the all-atom (blue), spline (green), and coarse-grained neural network models (red). **(e)** Representative Chignolin conformations in the three minima from (a–c) all-atom simulation and (a′-c′) coarse-grained neural network model. Reprinted with permission from Wang et al. (2019).

The parameterization of force fields and semiempirical quantum mechanics have also been performed integrating ML and evolutionary algorithms (Wang et al., 2019), which were successfully applied in MD (Wang et al., 2019). Constructing coarse-grained molecular models has been a common approach to extend the time/length-scales accessible to large or complex systems (Wang et al., 2019). These models have allowed establishing suitable interaction potentials for properties of high-resolution models or experimental data. Wang et al. (2019) have reformulated coarse-graining as a supervised machine learning problem, by using statistical learning theory for decoupling the coarse-graining error, and cross-validation for choosing and comparing the performance of distinct models. For that purpose, the authors developed a DL model, that learned coarse-grained free-energy functions and was trained by a force-matching strategy (see Figure 7).

The proposed framework automatically learned multiple terms necessary for accurate coarse-grained force fields, i.e., was able to keep relevant invariances and incorporate physics knowledge, avoiding the sampling of unphysical structures.

The class of coarse-grained directed neural networks can thus be trained with the force-matching principle and can encode all physically relevant invariances and constraints, including invariance of (i) the free-energy and mean force with respect to translation of the molecule, (ii) the free-energy and variance of the mean force associated to molecular rotation, and considering (iii) the mean force being a conservative force field generated by the free-energy, and (iv) a prior energy for preventing deviations of the simulations with coarse-grained neural networks into unphysical state space regions, i.e., states displaying overstretched bonds or clashing atoms, which are captured out of the training data.

The proposed strategy also outperformed classical coarse-graining approaches, which generally failed to capture relevant features of the free-energy surface, providing the all-atom explicit-solvent free-energy surfaces estimated with models including just a few coarse-grained beads, in the absence of solvent (Wang et al., 2019).

The integration of ML in MD simulations have also been useful for understanding the rate and yield of chemical reactions and providing key mechanistic details (Christensen et al., 2019; Häse et al., 2019). For instance, an unsupervised ML analysis tool based on Bayesian neural networks (BNNs) was proposed by Häse et al. (2019) to extract relevant information from *ab initio* MD simulation of chemical reactions (Häse et al., 2019). BNNs have been optimized to predict a specific outcome of an *ab initio* MD simulation corresponding to the dissociation time of the unmethylated and tetramethylated 1,2-dioxetane molecules, from the initial nuclear geometry and velocities. Predictions based on BNNs showed that an earlier dissociation was related to the planarization of the two formaldehyde moieties and also to the symmetric shortening of the C–O bonds, respecting the octet rule, i.e., the relation between bond order and bond length and orbital hybridization (Häse et al., 2019).

Rupp et al. (2012) have developed a ML algorithm based on non-linear statistical regression to predict the atomization energies of organic molecules. The proposed model employed a subset of seven thousand elements of the database, and a library of more than 100 stable and synthetically-tractable organic compounds. The target data used to train the model included atomization energies of the compounds calculated using the PBE0 hybrid functional. Cartesian coordinated and nuclear charge were used as descriptors in a “Coulomb” matrix representation. A mean-absolute error accuracy of 14.9 kcal/mol was achieved using a small fraction of the compounds for the training set. Similar accuracy, ca. 15.3 kcal/mol, was obtained considering an external validation set of 6,000 compounds showing the potential transferability of the model within in-class compounds. It was notable to outline QM-calculated energies, with a mean-absolute error of ca. 15 kcal/mol, without using the Schrodinger Equation in the ML algorithm. It was also suggested that the DLNs-based model should outperform the traditional ML-approach (Goh et al., 2017).

More recently, an alternative approach based ML algorithms for supplementing existing QM algorithms was proposed (Ramakrishnan et al., 2015). A Δ-learning approach, involving a ML correction term was developed. Such correction was used in DFT calculated properties for predicting the corresponding quantity at the G4MP2 level of theory. This combined QM/ML approach gathers approximate but fast legacy QM approximations and big-data based QM estimates, trained on results across chemical space, despite being applied using only traditional ML algorithms (Ramakrishnan et al., 2015).

Gómez-Bombarelli et al. (2018) have applied DL for generating and optimizing functional compounds, such as drug-like molecules. The proposed model allowed converting discrete representations of molecules from and into a multidimensional continuous representation, and generating new molecules for exploration and optimization.

A DLN was trained on a a large set of existing chemical structures to build an encoder, which converts the discrete representation of a molecule into a continuous vector, a decoder, that transforms the continuous vector into discrete molecular representations (e.g., SMILES string), and a predictor, which estimates chemical properties from the latent continuous vector representation of the molecule. These representations allowed generating new chemical structures automatically by employing simple operations in the latent space (e.g., decoding random vectors, perturbing defined chemical structures, and interpolating between molecules), and applying gradient-based optimization for a oriented-search of functional molecules (Gómez-Bombarelli et al., 2018).

DLNs have also been applied for exploring the molecular conformational space of proteins. Some authors (Degiacomi, 2019) have demonstrated that generative neural networks trained on protein structures, extracted from molecular simulation, can be employed to create new conformations complementing pre-existing ones. The model was trained and tested in a protein-protein docking scenario to account for specific motions occurring upon binding.

The fewer examples of DLNs applications in quantum chemistry suggest that it is still in an earlier stage of development compared to other approaches including computational structural biology and computer-aided drug design.

### Planning and Predicting Reactions and Routes

Some practical questions in organic chemistry have been addressed by ML approaches, including the identification of the most suitable synthesis method for a specific compound and the optimal conditions (reactants, solvent, catalyst, temperature, and among others) for ensuring region/chemo/stereo selectivity and obtaining the highest yields, estimating the precise rate, yield and time for the reaction, predicting major/minor product, and also evaluating similarity between reactions (Wei et al., 2016; Ahneman et al., 2018).

Making predictions in reactive chemical systems can also resort to DL. Segler and Waller (2017) and Segler et al. (2018) have predicted reaction rules considering fundamental substructures of reactants and products, allowing to return a product, given a reactant as input, and vice versa. In simple terms, a reaction rule is a pattern guiding the interaction process for a set of reactants and suggesting potential chemical products. As the knowledge available in often inaccurate, such rules are often ambiguous or even incomplete (Kishimoto et al., 2018). However, there are some successful examples, such as the recent outcomes of Chematica. Grzybowski et al. (2018) have assembled the relevant transformations that connect chemical species into a large network. The latter have codified and organized the known pathways through chemical space and displays nodes of molecules, elements and chemical reactions, collected by linking reactants to products on the basis of core reactions.

The Chematica platform comprises network theory, high-performance computing, artificial intelligence, and expert chemical knowledge to accelerate the design of synthetic pathways leading to new targets. However, the experimental verification of the respective predictions was carried out recently (Grzybowski et al., 2018). The authors have described the results of a systematic approach in which synthetic pathways leading to eight targets with distinct structures and of medicinal relevance were designed without human supervision and experimentally validated. There are other prominent products such as ChemPlanner, and Synthia created from databases of rules for chemical transformations. Both platforms incorporate ML algorithms and allows navigating through chemical space using those rules and suggesting to the user possible ways to synthesize a target molecule. Synthia also employs MD, quantum mechanics, and electronic properties to infer on the viability of a transformation and on the stability of an intermediate along a synthesis route (Klucznik et al., 2018).

Reaction prediction and retrosynthesis are the mainstays of organic chemistry. Retrosynthesis has been used for planning synthesis of small organic molecules, in which target molecules are recursively converted into progressively simpler precursors (Segler and Waller, 2017). However, the results obtained from the *in silico* version of this process are not, in general, adequate. Rule-based procedures have been extensively employed for solving, computationally, both reaction prediction and retrosynthesis. However, reactivity conflicts are often generated, since reaction rules tend to ignore the molecular context. It is often difficult to predict how a compound would behave in practice, unless an experiment is carried out (Granda et al., 2018). Evaluating a candidate sequence of reaction steps means that the synthesis of a given compound is also difficult. In chemical synthesis planning, Szymkuć et al. (2016) have discussed these issues. Segler and Waller have reported (Segler et al., 2018) that the prioritization of the most suitable conversion rules, as well as the approach to conflicting or complexity raising issues can be achieved by learning with DLNs. The authors have trained their model on ca. three million reactions, exhibiting accuracies of 97 and 95% for reaction prediction and retrosynthesis, respectively, on a validation set of ca. one million reactions. Following this procedure, the same authors have applied Monte Carlo tree search and symbolic artificial intelligence to find retrosynthetic routes. DLNs were trained on the whole set of published organic reactions (Segler et al., 2018).

Coley et al. (2017, 2018b) have performed DL with features based on the alterations of reactants and have determined scores for putative products. The product was modeled as a true target molecule (product) if it was generated by a reaction covered by the patent literature, and as a false product otherwise. More recently Coley et al. (2018b) have put forward a new definition addressing the synthetic complexity in order to compare with the expected number of reaction steps required for producing target molecules, with known compounds as reasonable starting materials. Specifically, a neural network model was trained on 12 million reactions from the Reaxys database, imposing a pairwise inequality constraint and showing that the products of published chemical reaction are, on average, more synthetically complex than their corresponding reactants.

A graph-link-prediction-based procedure was formulated by Savage et al. (2017) to predict candidate molecules (reactants), given a target molecule (product) as input and to discover adequate synthesis routes for producing the targets. This was employed over the Network of Organic Chemistry constructed from eight million chemical reactions described in the US patent literature in the 1976–2013 period (Savage et al., 2017). The proposed evaluation demonstrated that Factorization Machines, trained with chemistry-specific information, outperforms similarity-based methods of chemical structures. In these approaches, a fingerprint is built from a graphical representation of the molecule, containing the respective structural information and chemical features. The latter can be selected using different approaches (Morgan, 1965; Rogers and Hahn, 2010). Some neural graph fingerprints have displayed significant predictive performance (Duvenaud et al., 2015). The detection of molecular active substructures (e.g., a moiety impacting on a disease and a moiety that confers structural stability) can also be performed with ML (Duvenaud et al., 2015).

Researchers have also designed a chemical-handling robot for screening and predicting chemical reactivity using ML. The authors have found four novel reactions, demonstrating the respective potential in discovering reactions. Chemical reactions related to many different pathways can lead to a desired molecule. To find the best pathways, discovering new chemical reactivity is crucial to make the processes that produce chemicals, pharmaceuticals and materials more sustainable, environmentally-friendly and efficient. However, discovering new reactions is usually an unpredictable and time-consuming process that's constrained by a top-down approach involving expert knowledge to target a particular molecule.

Other researchers (Granda et al., 2018) have created an organic synthesis robotic ML system able to explore the reactivity several reagents from the bottom-up with no specific target. By performing ca. 10% of 969 possible reactions from a set of 18 reagents, the proposed system allowed predicting the reactivity of the remaining 90% of reactions with an accuracy of 86%. The database was continuously updated by performing multiple experiments based on the reactivity data collected. This allowed discovering new reactions that were inspected to isolate and characterize the new compounds (Granda et al., 2018).

### Supporting Analytical Chemistry and Catalysis

Analytical chemistry is possibly the area corresponding to the longest history, but also one that mostly displays embryonic applications of ML. A large number of statistical analyses and ML expert systems have been implemented in analytical chemistry for a long time (e.g., comparing and classifying mass spectra, NMR, or IR through assessments on available compounds) (Lipkowitz and Boyd, 1995; Mayer and Baeumner, 2019). Until recently, ML approaches were mainly employed to explain chemical reactions and to provide valuable predictive insights. Currently, it is possible to predict unexpected reactive outcomes, or relevant mechanistic insights for catalytic processes. A survey of some of these contributions can be found in Durand and Fey (2019).

Other groups (Ghosh et al., 2019) have proposed DL methods for predicting molecular excitation spectra. Considering the electronic density of the states of 132 k organic compounds, the authors have built three different neural network architectures: a multilayer perceptron (MLP), a convolutional neural network (CNN), and a DLNs. The coordinates and charge of the atoms in each molecule were used as inputs for the neural networks. The DLNs reached the best performance with a root-mean-square error (RMSE) of 0.19 eV, while MLP and CNN were able to learn spectra with a RMSE of 0.3 and 0.23 eV, respectively. Both CNN and DLNs allowed identifying subtle variations in the spectral shape. The structures of 10 k organic molecules previously unseen were scanned and the instant predictions on spectra were obtained to identify molecules for further applications (Ghosh et al., 2019).

A new computational approach, denoted as quantitative profile-profile relationship (QPPR) modeling, and based on ML techniques, has been proposed for predicting the pre-discharge chemical profiles of ammunition components from the components of the respective post-discharge gunshot residue (Gallidabino et al., 2019). The predicted profiles can be compared with other measured profiles to perform evidential associations in forensic investigations. Specifically, the approach was optimized and assessed for the prediction of GC-MS profiles of smokeless powders (SLPs) obtained from organic gunshot residues, considering nine ammunition types. A high degree of similarity between predicted and experimentally measured profiles was found, after applying 14 ML techniques, with a median correlation of 0.982 (Gallidabino et al., 2019). Receiver operating characteristic (ROC) analysis was employed to assess association performances, and allowed comparing predicted–predicted and predicted–measured profiles, producing areas under the curve (AUCs) of 0.976 and 0.824, respectively, in extrapolation mode. On the other hand, AUCs of 0.962 and 0.894 were obtained in the interpolation mode. These results were approximated to the values obtained from the comparison of the measured SLP profiles (AUC = 0.998), demonstrating excellent potential to correctly associate evidence in a number of different forensic situations (Gallidabino et al., 2019). The advantages of this approach are numerous and may be extended to other fields in analytical sciences that routinely experience mutable chemical signatures, including the analysis of explosive devices, toxicological samples and environmental pollutants (Gallidabino et al., 2019).

The integration of ML-based algorithms in a chemosensor has also pointed out the future of ML and the artificial internet of things applicability, i.e., optimized sensors, linked to a central data analysis unit via wireless (Mayer and Baeumner, 2019).

Additionally, researchers have used ML to develop tools for predicting catalytic components and dynamics. For instance, the identification and prediction of ligands for metal-catalyzed coupling reaction have been conducted for designing a synthetic economic and ecological route, with the potential to be expanded into a system of pharmaceutical interest (Durand and Fey, 2019). Durand and Fey have recently summarized calculations of several ligand descriptors, focusing on homogeneous organometallic catalysis. Different approaches for calculating steric and electronic parameters were also reviewed and assessed, and a set of descriptors for a wide range of ligands (e.g., 30 monodentate phosphorus (III) donor ligands, 23 bidentate P,P-donor ligands, and 30 carbenes) were collected.

Different case studies covering the application of these descriptors, including maps and models and DFT calculations, have been discussed, demonstrating the usefulness of descriptor-oriented studies of catalysis for guiding experiments and successfully evaluate and compare the proposed models (Durand and Fey, 2019).

Li and Eastgate (2019) have designed a ML-based tool for acting on transition metal-catalyzed carbon–nitrogen coupling reactions encompassing phosphine ligands, which are often involved in pharmaceutical syntheses. The data set of the system was composed of literature documents reporting coupling reactions with phosphine ligands. The input variables were the molecular features of ligand electrophiles and nucleophiles, and the phosphine ligands were de output obtained in successful reactions. The tools used substrate fingerprints, to build a multiclass predictive model and identify the ligands prone to function in a Pd-catalyzed C–N coupling reaction. The resulting probabilities were associated to the corresponding ligand (cPMIs) to determine a probability-weighted predicted holistic PMI for the transformation, considering the synthesis of the ligand. This novel ML approach were developed for estimating the probability of success for ligands, given specified electrophile and nucleophile combinations, illustrated in the a Pd-catalyzed C–N coupling context. The neural network allowed thus improving the predictive performance of the top-N accuracy over other ML approaches. Further application of this tool will foster the development of frameworks based on criteria-decision analytics, optimizing the design of manufacturing processes.

Designing catalysts using computational approaches is also a major challenge in chemistry. Conventional approaches have been restricted to calculate properties for a complex and large number of potential catalysts. More recently, innovative approaches for inverse design have emerged, for finding the desired property and optimizing the respective chemical structure. The chemical space has been explored by combining gradient-based optimization, alchemical transformations, and ML. These efforts have been duly reviewed in the context of inverse design and relevance to developing catalytic technologies (Freeze et al., 2019). These approaches have offered new opportunities for identifying catalysts using efficient methods that circumvent the need for high-throughput screening and reduce the array of compounds and materials displaying the target properties and can be experimentally validated. For instance, inverse design can be employed for modulating catalytic activity via alterations in the first and second coordination spheres of the catalyst binding site (e.g., functionality of catalytic cofactors in enzymes).

One possible approach to inverse design is to use the synthetic accessibility score, commonly used for drug molecules, in the scoring functions of inverse design for ensuring synthetic feasibility. For that purpose, empirical parameters can be used to describe molecules without the cost of using 3D coordinates for an entire structure and without using a model to describe the complex interactions from geometries.

The major progress on inverse design relies on optimization algorithms, which govern the process for exploring a specific space, improving identification rates of parameters that allows optimizing the value of the scoring function. For example, the Classical Optimal Control Optimization algorithm, used for global energy minimization, is based on the diffeomorphic modulation under the observable-response-preserving homotopy algorithm, and lead the classical dynamics of a probe particle, driven by an external field for reaching the optimal value of a multidimensional function, by adjusting iteratively field control parameters over the gradient of the scoring function related to the controls. However, the respective use for scoring functions in inverse design applications still remain a challenge (Freeze et al., 2019). Scoring functions allow correlating molecular descriptors to catalytic properties for finding catalysts via gradient-based optimization. In a simple example, similar molecules often display distinct catalytic activity due to subtle effects that must be detected by scoring functions. Such effects may be determined by combining experimentation to build adequate training sets of systems with different values of selected properties for determining feature sets able to detect such properties. ML can also be used to evaluate performance scores for GA-based methods.

The application of autoencoders have allowed transforming SMILES representations of compounds into a continuous latent space in order to optimize chemical properties, including synthetic accessibility score and Quantitative Estimation of Drug Likeness. Additionally, by resorting to gradient-based methods the latent space can be intersected to predict new candidate structures for being synthesized and tested.

The integration of inverse design, gradient-based optimization and ML is a very promising strategy to shorten the long path toward catalyst discovery (Freeze et al., 2019). Also, other related methods that have been implemented to scrutinize the chemical space for drug development can be applied for catalyst discovery, as described in Freeze et al. (2019).

## Concluding Remarks

This review has sought to provide a sample of ML approaches that support the major research trends in Chemistry, especially in computational chemistry, focusing on DLNs. Such an approaches have offered the possibility of solving chemical problems that cannot be described and explained via conventional methods. In the last few years, the application of ML to the optimization and prediction of molecular properties has become very popular, since more researchers are trained and acquired technical skills to develop and use such methods. ML applications are area-dependent and follow, in fact, a more or less obvious pattern. For instance, medicinal chemistry excels in structure-activity relationships. In other words, each sub-field is progressing essentially in activities that belong to its core subjects. It seems that these fields are evolving naturally, and we cannot identify significant disruptive trends.

Despite the historical route of ML methods involving the implementation of clustering or dimensionality reduction approaches, to provide a simple, low dimensional, or coarse-grained representations of structural and dynamical patterns of complex chemical systems, the interplay between innovative ML-driven predictions and molecular simulations can be combined to make time-consuming electronic calculations feasible, obtain accurate interatomic potentials on complex systems, and provide a rational design of molecules and materials. However, the convergence between different ML algorithms is a major challenge to achieve a definite progress in the chemistry fields.

Unsupervised learning may also contribute to elucidate the operating aspects of supervised algorithms, while supervised approaches may contribute to the formulation of objective metrics to evaluate the performance of unsupervised approaches.

In Chemistry DL is still at an incipient stage, particularly in computational chemistry, although the pace of contributions has been increasing very recently. One of the major challenges is the disparity, quality and interpretability of the generated outcomes. Paired with the sophistication and ability of GPU-accelerated computing for training DLNs and the massive growth of chemical information used for training DLNs, it is anticipated that DL algorithms will be an invaluable engine for computational chemistry. DL uses a hierarchical cascade of non-linear functions allowing to learn representations and capture the required features from raw chemical data, necessary for predicting target physicochemical properties.

Considering the recent effort on the topic, DL models have been implemented in various Chemistry sub-fields, including quantum-chemistry, compound and materials design, with superior performances to conventional ML algorithms. There is still tremendous room for improved model accuracy and interpretability. While industrial sectors will continue driving many advances, academia will continue playing a critical role in supplying innovative technical and practical contributions, as well as in fostering cross-disciplinary cooperation.

## Author Contributions

TC performed the bibliometrics analysis, collected the relevant studies in the context of the review, structured, and wrote the paper. AP directed the work, contributed to the interpretation of the data, and to the structure of the review. Both authors reviewed the manuscript.

### Conflict of Interest

The authors declare that the research was conducted in the absence of any commercial or financial relationships that could be construed as a potential conflict of interest.

## References

[B1] Agrafiotis D. K. Cedeño W. Lobanov V. S. (2002). On the use of neural network ensembles in QSAR and QSPR. J. Chem. Inf. Comput. Sci. 42, 903–911. 10.1021/ci0203702 12132892

[B2] Ahn S. Hong M. Sundararajan M. Ess D. H. Baik M.-H. (2019). Design and optimization of catalysts based on mechanistic insights derived from quantum chemical reaction modeling. Chem. Rev. 119, 6509–6560. 10.1021/acs.chemrev.9b00073 31066549

[B3] Ahneman D. T. Estrada J. G. Lin S. Dreher S. D. Doyle A. G. (2018). Predicting reaction performance in C–N cross-coupling using machine learning. Science 360, 186–190. 10.1126/science.aar5169 29449509

[B4] Alipanahi B. Delong A. Weirauch M. T. Frey B. J. (2015). Predicting the sequence specificities of DNA- and RNA-binding proteins by deep learning. Nat. Biotechnol. 33, 831–838. 10.1038/nbt.3300 26213851

[B5] Artrith N. Urban A. (2016). An implementation of artificial neural-network potentials for atomistic materials simulations: performance for TiO_2_. Comput. Mater. Sci. 114, 135–150. 10.1016/j.commatsci.2015.11.047

[B6] Arús-Pous J. Blaschke T. Ulander S. Reymond J.-L. Chen H. Engkvist O. (2019). Exploring the GDB-13 chemical space using deep generative models. J. Cheminform. 11:20. 10.1186/s13321-019-0341-z 30868314 PMC6419837

[B7] Aspuru-Guzik A. Baik M.-H. Balasubramanian S. Banerjee R. Bart S. Borduas-Dedekind N.. (2019). Charting a course for chemistry. Nat. Chem. 11, 286–294. 10.1038/s41557-019-0236-7 30903035

[B8] Bartók A. P. Kondor R. Csányi G. (2013). On representing chemical environments. Phys. Rev. B 87:184115 10.1103/PhysRevB.87.184115

[B9] Bartók A. P. Payne M. C. Kondor R. Csányi G. (2010). Gaussian approximation potentials: the accuracy of quantum mechanics, without the electrons. Phys. Rev. Lett. 104:136403. 10.1103/PhysRevLett.104.136403 20481899

[B10] Behler J. (2011a). Atom-centered symmetry functions for constructing high-dimensional neural network potentials. J. Chem. Phys. 134:074106. 10.1063/1.3553717 21341827

[B11] Behler J. (2011b). Neural network potential-energy surfaces in chemistry: a tool for large-scale simulations. Phys. Chem. Chem. Phys. 13, 17930–17955. 10.1039/c1cp21668f 21915403

[B12] Behler J. (2016). Perspective: machine learning potentials for atomistic simulations. J. Chem. Phys. 145:170901 10.1063/1.4966192 27825224

[B13] Behler J. Parrinello M. (2007). Generalized neural-network representation of high-dimensional potential-energy surfaces. Phys. Rev. Lett. 98:146401. 10.1103/PhysRevLett.98.146401 17501293

[B14] Benjamin S.-L. Carlos O. Gabriel L. G. Alan A.-G. (2017). Optimizing Distributions Over Molecular Space. An Objective-Reinforced Generative Adversarial Network for Inverse-design Chemistry (ORGANIC). ChemRxiv [Preprint]. 10.26434/chemrxiv.5309668.v3

[B15] Bose S. Dhawan D. Nandi S. Sarkar R. R. Ghosh D. (2018). Machine learning prediction of interaction energies in rigid water clusters. Phys. Chem. Chem. Phys. 20, 22987–22996. 10.1039/C8CP03138J 30156235

[B16] Botu V. Batra R. Chapman J. Ramprasad R. (2017). Machine learning force fields: construction, validation, and outlook. J. Phys. Chem. C 121, 511–522. 10.1021/acs.jpcc.6b10908

[B17] Botu V. Ramprasad R. (2015a). Adaptive machine learning framework to accelerate ab initio molecular dynamics. Int. J. Quantum Chem. 115, 1074–1083. 10.1002/qua.24836

[B18] Botu V. Ramprasad R. (2015b). Learning scheme to predict atomic forces and accelerate materials simulations. Phys. Rev. B 92:094306 10.1103/PhysRevB.92.094306

[B19] Brockherde F. Vogt L. Li L. Tuckerman M. E. Burke K. Müller K.-R. (2017). Bypassing the Kohn-Sham equations with machine learning. Nat. Commun. 8:872. 10.1038/s41467-017-00839-3 29021555 PMC5636838

[B20] Brown N. Fiscato M. Segler M. H. S. Vaucher A. C. (2019). GuacaMol: benchmarking models for *de novo* molecular design. J. Chem. Inf. Model. 59, 1096–1108. 10.1021/acs.jcim.8b00839 30887799

[B21] Butler K. T. Davies D. W. Cartwright H. Isayev O. Walsh A. (2018). Machine learning for molecular and materials science. Nature 559, 547–555. 10.1038/s41586-018-0337-2 30046072

[B22] Ceriotti M. (2019). Unsupervised machine learning in atomistic simulations, between predictions and understanding. J. Chem. Phys. 150:150901. 10.1063/1.5091842 31005087

[B23] Chakravarti S. K. (2018). Distributed representation of chemical fragments. ACS Omega 3, 2825–2836. 10.1021/acsomega.7b02045 30023852 PMC6044751

[B24] Chan H. Cherukara M. J. Narayanan B. Loeffler T. D. Benmore C. Gray S. K.. (2019). Machine learning coarse grained models for water. Nat. Commun. 10:379. 10.1038/s41467-018-08222-6 30670699 PMC6342926

[B25] Chandrasekaran A. Kamal D. Batra R. Kim C. Chen L. Ramprasad R. (2019). Solving the electronic structure problem with machine learning. NPJ Comput. Mater. 5:22 10.1038/s41524-019-0162-7

[B26] Chen H. Engkvist O. Wang Y. Olivecrona M. Blaschke T. (2018). The rise of deep learning in drug discovery. Drug Discov. Today 23, 1241–1250. 10.1016/j.drudis.2018.01.039 29366762

[B27] Chen M. Yu T.-Q. Tuckerman M. E. (2015). Locating landmarks on high-dimensional free energy surfaces. Proc. Natl. Acad. Sci. U.S.A. 112:3235. 10.1073/pnas.1418241112 25737545 PMC4371946

[B28] Chmiela S. Sauceda H. E. Müller K.-R. Tkatchenko A. (2018). Towards exact molecular dynamics simulations with machine-learned force fields. Nat. Commun. 9, 3887–3887. 10.1038/s41467-018-06169-2 30250077 PMC6155327

[B29] Chmiela S. Tkatchenko A. Sauceda H. E. Poltavsky I. Schütt K. T. Müller K.-R. (2017). Machine learning of accurate energy-conserving molecular force fields. Sci. Adv. 3:e1603015. 10.1126/sciadv.1603015 28508076 PMC5419702

[B30] Choi H. Kang H. Chung K.-C. Park H. (2019). Development and application of a comprehensive machine learning program for predicting molecular biochemical and pharmacological properties. Phys. Chem. Chem. Phys. 21, 5189–5199. 10.1039/C8CP07002D 30775759

[B31] Christensen A. S. Faber F. A. von Lilienfeld O. A. (2019). Operators in quantum machine learning: Response properties in chemical space. J. Chem. Phys. 150:064105. 10.1063/1.5053562 30769998

[B32] Chuang K. V. Keiser M. J. (2018a). Adversarial controls for scientific machine learning. ACS Chem. Biol. 13, 2819–2821. 10.1021/acschembio.8b00881 30336670

[B33] Chuang K. V. Keiser M. J. (2018b). Comment on “predicting reaction performance in C–N cross-coupling using machine learning”. Science 362:eaat8603 10.1126/science.aat8603 30442776

[B34] Coley C. W. Barzilay R. Jaakkola T. S. Green W. H. Jensen K. F. (2017). Prediction of organic reaction outcomes using machine learning. ACS Central Sci. 3, 434–443. 10.1021/acscentsci.7b00064 28573205 PMC5445544

[B35] Coley C. W. Green W. H. Jensen K. F. (2018a). Machine learning in computer-aided synthesis planning. Acc. Chem. Res. 51, 1281–1289. 10.1021/acs.accounts.8b00087 29715002

[B36] Coley C. W. Rogers L. Green W. H. Jensen K. F. (2018b). SCScore: synthetic complexity learned from a reaction corpus. J. Chem. Inf. Model. 58, 252–261. 10.1021/acs.jcim.7b00622 29309147

[B37] Cortés-Borda D. Kutonova K. V. Jamet C. Trusova M. E. Zammattio F. Truchet C. (2016). Optimizing the Heck–Matsuda reaction in flow with a constraint-adapted direct search algorithm. Organ. Process Res. Dev. 20, 1979–1987. 10.1021/acs.oprd.6b00310

[B38] Coveney Peter V. Dougherty Edward R. Highfield Roger R. (2016). Big data need big theory too. Philos. Trans. R. Soc. Math. Phys. Eng. Sci. 374:20160153. 10.1098/rsta.2016.0153 27698035 PMC5052735

[B39] Curtarolo S. Hart G. L. W. Nardelli M. B. Mingo N. Sanvito S. Levy O. (2013). The high-throughput highway to computational materials design. Nat. Mater. 12, 191–201. 10.1038/nmat3568 23422720

[B40] De S. Bartók A. P. Csányi G. Ceriotti M. (2016). Comparing molecules and solids across structural and alchemical space. Phys. Chem. Chem. Phys. 18, 13754–13769. 10.1039/C6CP00415F 27101873

[B41] Degiacomi M. T. (2019). Coupling molecular dynamics and deep learning to mine protein conformational space. Structure 27, 1034–1040.e1033. 10.1016/j.str.2019.03.018 31031199

[B42] Dral P. O. Von Lilienfeld O. A. Thiel W. (2015). Machine learning of parameters for accurate semiempirical quantum chemical calculations. J. Chem. Theory Comput. 11, 2120–2125. 10.1021/acs.jctc.5b00141 26146493 PMC4479612

[B43] Durand D. J. Fey N. (2019). Computational ligand descriptors for catalyst design. Chem. Rev. 119, 6561–6594. 10.1021/acs.chemrev.8b00588 30802036

[B44] Duvenaud D. K. Maclaurin D. Iparraguirre J. Bombarell R. Hirzel T. Aspuru-Guzik A. (2015). Convolutional networks on graphs for learning molecular fingerprints, in Advances in Neural Information Processing Systems, eds Cortes C. Lawrence N. D. Lee D. D Sugiyama M. Garnett R. (Montreal, QC), 2224–2232.

[B45] Ekins S. (2016). The next Era: deep learning in pharmaceutical research. Pharm. Res. 33, 2594–2603. 10.1007/s11095-016-2029-7 27599991 PMC5042864

[B46] El-Atta A. H. A. Hassanien A. E. (2017). Two-class support vector machine with new kernel function based on paths of features for predicting chemical activity. Inf. Sci. 403–404, 42–54. 10.1016/j.ins.2017.04.003

[B47] Elton D. C. Boukouvalas Z. Butrico M. S. Fuge M. D. Chung P. W. (2018). Applying machine learning techniques to predict the properties of energetic materials. Sci. Rep. 8:9059. 10.1038/s41598-018-27344-x 29899464 PMC5998124

[B48] Elton D. C. Boukouvalas Z. Fuge M. D. Chung P. W. (2019). Deep learning for molecular design—a review of the state of the art. Mol. Syst. Design Eng. 4, 828–849. 10.1039/C9ME00039A

[B49] Estrada J. G. Ahneman D. T. Sheridan R. P. Dreher S. D. Doyle A. G. (2018). Response to comment on “predicting reaction performance in C–N cross-coupling using machine learning”. Science 362:eaat8763 10.1126/science.aat8763 30442777

[B50] Faber F. A. Hutchison L. Huang B. Gilmer J. Schoenholz S. S. Dahl G. E.. (2017). Prediction errors of molecular machine learning models lower than hybrid DFT error. J. Chem. Theory Comput. 13, 5255–5264. 10.1021/acs.jctc.7b00577 28926232

[B51] Faber F. A. Lindmaa A. Von Lilienfeld O. A. Armiento R. (2016). Machine learning energies of 2 million elpasolite (ABC$_2$D$_6$) crystals. Phys. Rev. Lett. 117:135502. 10.1103/PhysRevLett.117.135502 27715098

[B52] Fleming N. (2018). How artificial intelligence is changing drug discovery. Nature 557, S55–S55. 10.1038/d41586-018-05267-x 29849160

[B53] Freeze J. G. Kelly H. R. Batista V. S. (2019). Search for catalysts by inverse design: artificial intelligence, mountain climbers, and alchemists. Chem. Rev. 119, 6595–6612. 10.1021/acs.chemrev.8b00759 31059236

[B54] Fuchs J.-A. Grisoni F. Kossenjans M. Hiss J. A. Schneider G. (2018). Lipophilicity prediction of peptides and peptide derivatives by consensus machine learning. Medchemcomm 9, 1538–1546. 10.1039/C8MD00370J 30288227 PMC6151477

[B55] Gallidabino M. D. Barron L. P. Weyermann C. Romolo F. S. (2019). Quantitative profile–profile relationship (QPPR) modelling: a novel machine learning approach to predict and associate chemical characteristics of unspent ammunition from gunshot residue (GSR). Analyst 144, 1128–1139. 10.1039/C8AN01841C 30474092

[B56] Gao H. Struble T. J. Coley C. W. Wang Y. Green W. H. Jensen K. F. (2018). Using machine learning to predict suitable conditions for organic reactions. ACS Central Sci. 4, 1465–1476. 10.1021/acscentsci.8b00357 30555898 PMC6276053

[B57] Gasteiger J. Zupan J. (1993). Neural networks in chemistry. Angew. Chem. Int. Ed. Eng. 32, 503–527. 10.1002/anie.199305031

[B58] Ghosh K. Stuke A. Todorović M. Jørgensen P. B. Schmidt M. N. Vehtari A.. (2019). Deep learning spectroscopy: neural networks for molecular excitation spectra. Adv. Sci. 6:1801367. 10.1002/advs.201801367 31065514 PMC6498126

[B59] Goh G. B. Hodas N. O. Vishnu A. (2017). Deep learning for computational chemistry. J. Comput. Chem. 38, 1291–1307. 10.1002/jcc.24764 28272810

[B60] Gómez-Bombarelli R. Wei J. N. Duvenaud D. Hernández-Lobato J. M. Sánchez-Lengeling B. Sheberla D.. (2018). Automatic chemical design using a data-driven continuous representation of molecules. ACS Central Sci. 4, 268–276. 10.1021/acscentsci.7b00572 29532027 PMC5833007

[B61] Granda J. M. Donina L. Dragone V. Long D.-L. Cronin L. (2018). Controlling an organic synthesis robot with machine learning to search for new reactivity. Nature 559, 377–381. 10.1038/s41586-018-0307-8 30022133 PMC6223543

[B62] Green C. P. Engkvist O. Pairaudeau G. (2018). The convergence of artificial intelligence and chemistry for improved drug discovery. Future Med. Chem. 10, 2573–2576. 10.4155/fmc-2018-0161 30499699

[B63] Griffen E. J. Dossetter A. G. Leach A. G. Montague S. (2018). Can we accelerate medicinal chemistry by augmenting the chemist with Big Data and artificial intelligence? Drug Discov. Today 23, 1373–1384. 10.1016/j.drudis.2018.03.011 29577971

[B64] Grisoni F. Neuhaus C. S. Gabernet G. Müller A. T. Hiss J. A. Schneider G. (2018). Designing anticancer peptides by constructive machine learning. ChemMedChem 13, 1300–1302. 10.1002/cmdc.201800204 29679519

[B65] Gromski P. S. Henson A. B. Granda J. M. Cronin L. (2019). How to explore chemical space using algorithms and automation. Nat. Rev. Chem. 3, 119–128. 10.1038/s41570-018-0066-y

[B66] Grzybowski B. A. Szymkuć S. Gajewska E. P. Molga K. Dittwald P. Wołos A. (2018). Chematica: a story of computer code that started to think like a chemist. Chem 4, 390–398. 10.1016/j.chempr.2018.02.024

[B67] Gupta A. Müller A. T. Huisman B. J. H. Fuchs J. A. Schneider P. Schneider G. (2018). Generative recurrent networks for *de novo* drug design. Mol. Inform. 37:1700111 10.1002/minf.201700111 PMC5836943 29095571

[B68] Hansen K. Biegler F. Ramakrishnan R. Pronobis W. Von Lilienfeld O. A. Müller K.-R.. (2015). Machine learning predictions of molecular properties: accurate many-body potentials and nonlocality in chemical space. J. Phys. Chem. Lett. 6, 2326–2331. 10.1021/acs.jpclett.5b00831 26113956 PMC4476293

[B69] Hansen K. Montavon G. Biegler F. Fazli S. Rupp M. Scheffler M.. (2013). Assessment and validation of machine learning methods for predicting molecular atomization energies. J. Chem. Theory Comput. 9, 3404–3419. 10.1021/ct400195d 26584096

[B70] Harel S. Radinsky K. (2018). Prototype-based compound discovery using deep generative models. Mol. Pharm. 15, 4406–4416. 10.1021/acs.molpharmaceut.8b00474 30063142

[B71] Häse F. Fdez. Galván I. Aspuru-Guzik A. Lindh R. Vacher M. (2019). How machine learning can assist the interpretation of ab initio molecular dynamics simulations and conceptual understanding of chemistry. Chem. Sci. 10, 2298–2307. 10.1039/C8SC04516J 30881655 PMC6385677

[B72] Häse F. Roch L. M. Aspuru-Guzik A. (2018). Chimera: enabling hierarchy based multi-objective optimization for self-driving laboratories. Chem. Sci. 9, 7642–7655. 10.1039/C8SC02239A 30393525 PMC6182568

[B73] He Y. Cubuk E. D. Allendorf M. D. Reed E. J. (2018). Metallic metal–organic frameworks predicted by the combination of machine learning methods and Ab initio calculations. J. Phys. Chem. Lett. 9, 4562–4569. 10.1021/acs.jpclett.8b01707 30052453

[B74] Hegde G. Bowen R. C. (2017). Machine-learned approximations to density functional theory hamiltonians. Sci. Rep. 7:42669. 10.1038/srep42669 28198471 PMC5309850

[B75] Hiller S. A. Golender V. E. Rosenblit A. B. Rastrigin L. A. Glaz A. B. (1973). Cybernetic methods of drug design. I. Statement of the problem—the perceptron approach. Comput. Biomed. Res. 6, 411–421. 10.1016/0010-4809(73)90074-8 4747104

[B76] Houben C. Lapkin A. A. (2015). Automatic discovery and optimization of chemical processes. Curr. Opin. Chem. Eng. 9, 1–7. 10.1016/j.coche.2015.07.001

[B77] Houben C. Peremezhney N. Zubov A. Kosek J. Lapkin A. A. (2015). Closed-loop multitarget optimization for discovery of new emulsion polymerization recipes. Organ. Process Res. Dev. 19, 1049–1053. 10.1021/acs.oprd.5b00210 26435638 PMC4579860

[B78] Huang S.-D. Shang C. Kang P.-L. Liu Z.-P. (2018). Atomic structure of boron resolved using machine learning and global sampling. Chem. Sci. 9, 8644–8655. 10.1039/C8SC03427C 30627388 PMC6289100

[B79] Hughes Z. E. Thacker J. C. R. Wilson A. L. Popelier P. L. A. (2019). Description of potential energy surfaces of molecules using FFLUX machine learning models. J. Chem. Theory Comput. 15, 116–126. 10.1021/acs.jctc.8b00806 30507180

[B80] Iype E. Urolagin S. (2019). Machine learning model for non-equilibrium structures and energies of simple molecules. J. Chem. Phys. 150:024307. 10.1063/1.5054968 30646726

[B81] Janet J. P. Chan L. Kulik H. J. (2018). Accelerating chemical discovery with machine learning: simulated evolution of spin crossover complexes with an artificial neural network. J. Phys. Chem. Lett. 9, 1064–1071. 10.1021/acs.jpclett.8b00170 29425453

[B82] Jaquis B. J. Li A. Monnier N. D. Sisk R. G. Acree W. E. Lang A. S. (2019). Using machine learning to predict enthalpy of solvation. J. Solution Chem. 48, 564–573. 10.1007/s10953-019-00867-1

[B83] Jensen J. H. (2019). A graph-based genetic algorithm and generative model/Monte Carlo tree search for the exploration of chemical space. Chem. Sci. 10, 3567–3572. 10.1039/C8SC05372C 30996948 PMC6438151

[B84] Jha D. Ward L. Paul A. Liao W.-K. Choudhary A. Wolverton C.. (2018). ElemNet: deep learning the chemistry of materials from only elemental composition. Sci. Rep. 8:17593. 10.1038/s41598-018-35934-y 30514926 PMC6279928

[B85] Jørgensen P. B. Mesta M. Shil S. Lastra J. M. G. Jacobsen K. W. Thygesen K. S.. (2018a). Machine learning-based screening of complex molecules for polymer solar cells. J. Chem. Phys. 148:241735. 10.1063/1.5023563 29960358

[B86] Jørgensen P. B. Schmidt M. N. Winther O. (2018b). Deep generative models for molecular science. Mol. Inform. 37:1700133. 10.1002/minf.201700133 29405647

[B87] Kadurin A. Nikolenko S. Khrabrov K. Aliper A. Zhavoronkov A. (2017). druGAN: an advanced generative adversarial autoencoder model for *de novo* generation of new molecules with desired molecular properties *in silico*. Mol. Pharm. 14, 3098–3104. 10.1021/acs.molpharmaceut.7b00346 28703000

[B88] Kanamori K. Toyoura K. Honda J. Hattori K. Seko A. Karasuyama M. (2018). Exploring a potential energy surface by machine learning for characterizing atomic transport. Phys. Rev. B 97:125124 10.1103/PhysRevB.97.125124

[B89] Kang S. Cho K. (2018). Conditional molecular design with deep generative models. J. Chem. Inf. Model. 59, 43–52. 10.1021/acs.jcim.8b00263 30016587

[B90] Kang X. Zhao Y. Li J. (2018). Predicting refractive index of ionic liquids based on the extreme learning machine (ELM) intelligence algorithm. J. Mol. Liq. 250, 44–49. 10.1016/j.molliq.2017.11.166

[B91] Keil M. Exner T. E. Brickmann J. (2004). Pattern recognition strategies for molecular surfaces: III. Binding site prediction with a neural network. J. Comput. Chem. 25, 779–789. 10.1002/jcc.10361 15011250

[B92] Kishimoto A. Buesser B. Botea A. (2018). AI meets chemistry, in Thirty-Second AAAI Conference on Artificial Intelligence. Ireland: IBM Research.

[B93] Klucznik T. Mikulak-Klucznik B. Mccormack M. P. Lima H. Szymkuć S. Bhowmick M. (2018). Efficient syntheses of diverse, medicinally relevant targets planned by computer and executed in the laboratory. Chem 4, 522–532. 10.1016/j.chempr.2018.02.002

[B94] Kowalik M. Gothard C. M. Drews A. M. Gothard N. A. Weckiewicz A. Fuller P. E.. (2012). Parallel optimization of synthetic pathways within the network of organic chemistry. Angew. Chem. Int. Ed. 51, 7928–7932. 10.1002/anie.201202209 22807100

[B95] Krallinger M. Rabal O. Lourenço A. Oyarzabal J. Valencia A. (2017). Information retrieval and text mining technologies for chemistry. Chem. Rev. 117, 7673–7761. 10.1021/acs.chemrev.6b00851 28475312

[B96] Lecun Y. Bengio Y. Hinton G. (2015). Deep learning. Nature 521:436. 10.1038/nature14539 26017442

[B97] Lee A. A. Yang Q. Bassyouni A. Butler C. R. Hou X. Jenkinson S.. (2019). Ligand biological activity predicted by cleaning positive and negative chemical correlations. Proc. Natl. Acad. Sci. U.S.A. 116:3373. 10.1073/pnas.1810847116 30808733 PMC6397557

[B98] Li H. Collins C. R. Ribelli T. G. Matyjaszewski K. Gordon G. J. Kowalewski T. (2018a). Tuning the molecular weight distribution from atom transfer radical polymerization using deep reinforcement learning. Mol. Syst. Design Eng. 3, 496–508. 10.1039/C7ME00131B

[B99] Li H. Zhang Z. Liu Z. (2017). Application of artificial neural networks for catalysis: a review. Catalysts 7:306 10.3390/catal7100306

[B100] Li J. Eastgate M. D. (2019). Making better decisions during synthetic route design: leveraging prediction to achieve greenness-by-design. React. Chem. Eng. 4, 1595–1607. 10.1039/C9RE00019D

[B101] Li Y. Zhang L. Liu Z. (2018b). Multi-objective *de novo* drug design with conditional graph generative model. J. Cheminform. 10:33. 10.1186/s13321-018-0287-6 30043127 PMC6057868

[B102] Lipkowitz K. B. Boyd D. B. (1995). Reviews in Computational Chemistry 6. New York, NY: Wiley Online Library 10.1002/9780470125830

[B103] Lo Y.-C. Rensi S. E. Torng W. Altman R. B. (2018). Machine learning in chemoinformatics and drug discovery. Drug Discov. Today 23, 1538–1546. 10.1016/j.drudis.2018.05.010 29750902 PMC6078794

[B104] Mansbach R. A. Ferguson A. L. (2015). Machine learning of single molecule free energy surfaces and the impact of chemistry and environment upon structure and dynamics. J. Chem. Phys. 142:105101. 10.1063/1.4914144 25770561

[B105] Marques M. R. G. Wolff J. Steigemann C. Marques M. A. L. (2019). Neural network force fields for simple metals and semiconductors: construction and application to the calculation of phonons and melting temperatures. Phys.istry Chem. Phys. 21, 6506–6516. 10.1039/C8CP05771K 30843548

[B106] Mater A. C. Coote M. L. (2019). Deep learning in chemistry. J. Chem. Inf. Model. 59, 2545–2559. 10.1021/acs.jcim.9b00266 31194543

[B107] Matsuzaka Y. Uesawa Y. (2019). Optimization of a deep-learning method based on the classification of images generated by parameterized deep snap a novel molecular-image-input technique for quantitative structure-activity relationship (QSAR) analysis. Front. Bioeng. Biotechnol. 7, 65–65. 10.3389/fbioe.2019.00065 30984753 PMC6447703

[B108] Mayer M. Baeumner A. J. (2019). A megatrend challenging analytical chemistry: biosensor and chemosensor concepts ready for the internet of things. Chem. Rev. 119, 7996–8027. 10.1021/acs.chemrev.8b00719 31070892

[B109] Merk D. Grisoni F. Friedrich L. Schneider G. (2018). Tuning artificial intelligence on the *de novo* design of natural-product-inspired retinoid X receptor modulators. Commun. Chem. 1:68 10.1038/s42004-018-0068-1

[B110] Mezei P. D. Von Lilienfeld O. A. (2019). Non-covalent quantum machine learning corrections to density functionals. arXiv [preprint]. arXiv:1903.09010. 10.1021/acs.jctc.0c00181 32130000

[B111] Microsoft (2018). Machine Learning, Data Mining and Rethinking Knowledge at KDD 2018. London, UK: Microsoft.

[B112] Miller T. H. Gallidabino M. D. Macrae J. I. Hogstrand C. Bury N. R. Barron L. P.. (2018). Machine learning for environmental toxicology: a call for integration and innovation. Environ. Sci. Technol. 52, 12953–12955. 10.1021/acs.est.8b05382 30338686

[B113] Min K. Choi B. Park K. Cho E. (2018). Machine learning assisted optimization of electrochemical properties for Ni-rich cathode materials. Sci. Rep. 8:15778. 10.1038/s41598-018-34201-4 30361533 PMC6202356

[B114] Mitchell J. B. O. (2014). Machine learning methods in chemoinformatics. Wiley interdisciplinary reviews. Comput. Mol. Sci. 4, 468–481. 10.1002/wcms.1183 25285160 PMC4180928

[B115] Mitchell T. M. (1997). Machine Learning. Burr Ridge, IL: McGraw Hill.

[B116] Molga K. Dittwald P. Grzybowski B. A. (2019). Navigating around patented routes by preserving specific motifs along computer-planned retrosynthetic pathways. Chem 5, 460–473. 10.1016/j.chempr.2018.12.004

[B117] Montavon G. Rupp M. Gobre V. Vazquez-Mayagoitia A. Hansen K. Tkatchenko A. (2013). Machine learning of molecular electronic properties in chemical compound space. New J. Phys. 15:095003 10.1088/1367-2630/15/9/095003

[B118] Morawietz T. Singraber A. Dellago C. Behler J. (2016). How van der Waals interactions determine the unique properties of water. Proc. Natl. Acad. Sci. U.S.A. 113, 8368–8373. 10.1073/pnas.1602375113 27402761 PMC4968748

[B119] Morgan H. L. (1965). The generation of a unique machine description for chemical structures-a technique developed at chemical abstracts service. J. Chem. Doc. 5, 107–113. 10.1021/c160017a018

[B120] Müller A. T. Hiss J. A. Schneider G. (2018). Recurrent neural network model for constructive peptide design. J. Chem. Inf. Model. 58, 472–479. 10.1021/acs.jcim.7b00414 29355319

[B121] Nouira A. Crivello J.-C. Sokolovska N. (2018). CrystalGAN: learning to discover crystallographic structures with generative adversarial networks. arXiv [preprint]. arXiv:1810.11203.

[B122] Panteleev J. Gao H. Jia L. (2018). Recent applications of machine learning in medicinal chemistry. Bioorgan. Med. Chem. Lett. 28, 2807–2815. 10.1016/j.bmcl.2018.06.046 30122222

[B123] Popova M. Isayev O. Tropsha A. (2018). Deep reinforcement learning for *de novo* drug design. Sci. Adv. 4:eaap7885. 10.1126/sciadv.aap7885 30050984 PMC6059760

[B124] Pronobis W. Schütt K. T. Tkatchenko A. Müller K.-R. (2018). Capturing intensive and extensive DFT/TDDFT molecular properties with machine learning. Eur. Phys. J. B 91:178 10.1140/epjb/e2018-90148-y

[B125] Ramakrishnan R. Dral P. O. Rupp M. Von Lilienfeld O. A. (2014). Quantum chemistry structures and properties of 134 kilo molecules. Sci. Data 1:140022. 10.1038/sdata.2014.22 25977779 PMC4322582

[B126] Ramakrishnan R. Dral P. O. Rupp M. Von Lilienfeld O. A. (2015). Big data meets quantum chemistry approximations: the Δ-machine learning approach. J. Chem. Theory Comput. 11, 2087–2096. 10.1021/acs.jctc.5b00099 26574412

[B127] Ramakrishnan R. Von Lilienfeld O. A. (2017). Machine learning, quantum chemistry, and chemical space, in Reviews in Computational Chemistry, Vol. 30, eds Parrill A. L. Lipkowitz K. B. (Wiley), 225–256. 10.1002/9781119356059.ch5

[B128] Richmond C. J. Miras H. N. De La Oliva A. R. Zang H. Sans V. Paramonov L.. (2012). A flow-system array for the discovery and scale up of inorganic clusters. Nat. Chem. 4, 1037–1043. 10.1038/nchem.1489 23174985

[B129] Rogers D. Hahn M. (2010). Extended-connectivity fingerprints. J. Chem. Inf. Model. 50, 742–754. 10.1021/ci100050t 20426451

[B130] Rupp M. (2015). Machine learning for quantum mechanics in a nutshell. Int. J. Quantum Chem. 115, 1058–1073. 10.1002/qua.24954

[B131] Rupp M. Ramakrishnan R. Von Lilienfeld O. A. (2015). Machine learning for quantum mechanical properties of atoms in molecules. J. Phys. Chem. Lett. 6, 3309–3313. 10.1021/acs.jpclett.5b01456

[B132] Rupp M. Tkatchenko A. Müller K.-R. Von Lilienfeld O. A. (2012). Fast and accurate modeling of molecular atomization energies with machine learning. Phys. Rev. Lett. 108:058301. 10.1103/PhysRevLett.108.058301 22400967

[B133] Sadowski P. Fooshee D. Subrahmanya N. Baldi P. (2016). Synergies between quantum mechanics and machine learning in reaction prediction. J. Chem. Inf. Model. 56, 2125–2128. 10.1021/acs.jcim.6b00351 27749058

[B134] Samuel A. L. (1959). Some studies in machine learning using the game of checkers. IBM J. Res. Dev. 3, 210–229. 10.1147/rd.33.0210

[B135] Sánchez-Lengeling B. Aspuru-Guzik A. (2017). Learning more, with less. ACS Central Sci. 3, 275–277. 10.1021/acscentsci.7b00153 28470043 PMC5408330

[B136] Sanchez-Lengeling B. Aspuru-Guzik A. (2018). Inverse molecular design using machine learning: generative models for matter engineering. Science 361, 360–365. 10.1126/science.aat2663 30049875

[B137] Sanchez-Lengeling B. Roch L. M. Perea J. D. Langner S. Brabec C. J. Aspuru-Guzik A. (2019). A Bayesian approach to predict solubility parameters. Adv. Theory Simul. 2:1800069 10.1002/adts.201800069

[B138] Savage J. Kishimoto A. Buesser B. Diaz-Aviles E. Alzate C. (2017). Chemical reactant recommendation using a network of organic chemistry, in Proceedings of the Eleventh ACM Conference on Recommender Systems (New York, NY: ACM), 210–214.

[B139] Schleder G. R. Padilha A. C. M. Acosta C. M. Costa M. Fazzio A. (2019). From DFT to machine learning: recent approaches to materials science–a review. J. Phys. Mater. 2:032001 10.1088/2515-7639/ab084b

[B140] Schneider G. (2018). Generative models for artificially-intelligent molecular design. Mol. Inform. 37:1880131. 10.1002/minf.201880131 29442446

[B141] Schütt K. Glawe H. Brockherde F. Sanna A. Müller K. Gross E. (2014). How to represent crystal structures for machine learning: towards fast prediction of electronic properties. Phys. Rev. B 89:205118 10.1103/PhysRevB.89.205118

[B142] Segler M. H. S. Preuss M. Waller M. P. (2018). Planning chemical syntheses with deep neural networks and symbolic AI. Nature 555, 604–610. 10.1038/nature25978 29595767

[B143] Segler M. H. S. Waller M. P. (2017). Neural-symbolic machine learning for retrosynthesis and reaction prediction. Chem. A Eur. J. 23, 5966–5971. 10.1002/chem.201605499 28134452

[B144] Shen X. Zhang T. Broderick S. Rajan K. (2018). Correlative analysis of metal organic framework structures through manifold learning of Hirshfeld surfaces. Mol. Syst. Design Eng. 3, 826–838. 10.1039/C8ME00014J

[B145] Simões R. S. Maltarollo V. G. Oliveira P. R. Honorio K. M. (2018). Transfer and multi-task learning in QSAR modeling: advances and challenges. Front. Pharmacol. 9:74. 10.3389/fphar.2018.00074 29467659 PMC5807924

[B146] Smith C. J. Nikbin N. Ley S. V. Lange H. Baxendale I. R. (2011). A fully automated, multistep flow synthesis of 5-amino-4-cyano-1,2,3-triazoles. Organ. Biomol. Chem. 9, 1938–1947. 10.1039/c0ob00815j 21283874

[B147] Smith J. S. Isayev O. Roitberg A. E. (2017). ANI-1: an extensible neural network potential with DFT accuracy at force field computational cost. Chem. Sci. 8, 3192–3203. 10.1039/C6SC05720A 28507695 PMC5414547

[B148] Smith J. S. Nebgen B. Lubbers N. Isayev O. Roitberg A. E. (2018a). Less is more: Sampling chemical space with active learning. J. Chem. Phys. 148:241733. 10.1063/1.5023802 29960353

[B149] Smith J. S. Roitberg A. E. Isayev O. (2018b). Transforming computational drug discovery with machine learning and AI. ACS Med. Chem. Lett. 9, 1065–1069. 10.1021/acsmedchemlett.8b00437 30429945 PMC6231187

[B150] Snyder J. C. Rupp M. Hansen K. Müller K.-R. Burke K. (2012). Finding density functionals with machine learning. Phys. Rev. Lett. 108:253002. 10.1103/PhysRevLett.108.253002 23004593

[B151] Stein H. S. Guevarra D. Newhouse P. F. Soedarmadji E. Gregoire J. M. (2019a). Machine learning of optical properties of materials – predicting spectra from images and images from spectra. Chem. Sci. 10, 47–55. 10.1039/C8SC03077D 30746072 PMC6334722

[B152] Stein H. S. Soedarmadji E. Newhouse P. F. Dan G. Gregoire J. M. (2019b). Synthesis, optical imaging, and absorption spectroscopy data for 179072 metal oxides. Sci. Data 6:9. 10.1038/s41597-019-0019-4 30918263 PMC6437643

[B153] Stevens J. G. Bourne R. A. Twigg M. V. Poliakoff M. (2010). Real-time product switching using a twin catalyst system for the hydrogenation of furfural in supercritical CO_2_. Angew. Chem. Int. Ed. 49, 8856–8859. 10.1002/anie.201005092 20928878

[B154] Szymkuć S. Gajewska E. P. Klucznik T. Molga K. Dittwald P. Startek M.. (2016). Computer-assisted synthetic planning: the end of the beginning. Angew. Chem. Int. Ed. 55:5904. 10.1002/anie.201506101 27062365

[B155] Thomsen J. U. Meyer B. (1989). Pattern recognition of the 1H NMR spectra of sugar alditols using a neural network. J. Magnetic Reson. 84, 212–217. 10.1016/0022-2364(89)90021-8

[B156] Varnek A. Baskin I. (2012). Machine learning methods for property prediction in chemoinformatics: quo vadis? J. Chem. Inf. Model. 52, 1413–1437. 10.1021/ci200409x 22582859

[B157] Venkatasubramanian V. (2019). The promise of artificial intelligence in chemical engineering: is it here, finally? AIChE J. 65, 466–478. 10.1002/aic.16489

[B158] Wang J. Olsson S. Wehmeyer C. Pérez A. Charron N. E. De Fabritiis G.. (2019). Machine learning of coarse-grained molecular dynamics force fields. ACS Central Sci. 5, 755–767. 10.1021/acscentsci.8b00913 31139712 PMC6535777

[B159] Ward L. Wolverton C. (2017). Atomistic calculations and materials informatics: a review. Curr. Opin. Solid State Mater. Sci. 21, 167–176. 10.1016/j.cossms.2016.07.002

[B160] Wei J. N. Duvenaud D. Aspuru-Guzik A. (2016). Neural networks for the prediction of organic chemistry reactions. ACS Central Sci. 2, 725–732. 10.1021/acscentsci.6b00219 27800555 PMC5084081

[B161] Welborn M. Cheng L. Miller T. F. (2018). Transferability in machine learning for electronic structure via the molecular orbital basis. J. Chem. Theory Comput. 14, 4772–4779. 10.1021/acs.jctc.8b00636 30040892

[B162] White D. Wilson R. C. (2010). Generative models for chemical structures. J. Chem. Inf. Model. 50, 1257–1274. 10.1021/ci9004089 20666408

[B163] Wu Y. Wang G. (2018). Machine learning based toxicity prediction: from chemical structural description to transcriptome analysis. Int. J. Mol. Sci. 19:2358. 10.3390/ijms19082358 30103448 PMC6121588

[B164] Wu Z. Ramsundar B. Feinberg E. N. Gomes J. Geniesse C. Pappu A. S. (2017). MoleculeNet: a benchmark for molecular machine learning. arXiv e-prints. Available online at: https://ui.adsabs.harvard.edu/abs/2017arXiv170300564W (accessed March 01, 2017). 10.1039/c7sc02664a PMC5868307 29629118

[B165] Xia R. Kais S. (2018). Quantum machine learning for electronic structure calculations. Nat. Commun. 9:4195. 10.1038/s41467-018-06598-z 30305624 PMC6180079

[B166] Xu Y. Lin K. Wang S. Wang L. Cai C. Song C.. (2019). Deep learning for molecular generation. Future Med. Chem. 11, 567–597. 10.4155/fmc-2018-0358 30698019

[B167] Zaspel P. Huang B. Harbrecht H. Von Lilienfeld O. A. (2019). Boosting quantum machine learning models with a multilevel combination technique: pople diagrams revisited. J. Chem. Theory Comput. 15, 1546–1559. 10.1021/acs.jctc.8b00832 30516999

[B168] Zhang P. Shen L. Yang W. (2019). Solvation free energy calculations with quantum mechanics/molecular mechanics and machine learning models. J. Phys. Chem. B 123, 901–908. 10.1021/acs.jpcb.8b11905 30557020 PMC6448400

[B169] Zhou Z. Kearnes S. Li L. Zare R. N. Riley P. (2018). Optimization of molecules via deep reinforcement learning. arXiv preprint arXiv:1810.08678. 10.1038/s41598-019-47148-x 31341196 PMC6656766

[B170] Zhou Z. Li X. Zare R. N. (2017). Optimizing chemical reactions with deep reinforcement learning. ACS Central Sci. 3, 1337–1344. 10.1021/acscentsci.7b00492 29296675 PMC5746857

[B171] Zielinski F. Maxwell P. I. Fletcher T. L. Davie S. J. Di Pasquale N. Cardamone S.. (2017). Geometry optimization with machine trained topological atoms. Sci. Rep. 7:12817. 10.1038/s41598-017-12600-3 28993674 PMC5634454

